# Epi-SSA: A novel epistasis detection method based on a multi-objective sparrow search algorithm

**DOI:** 10.1371/journal.pone.0311223

**Published:** 2024-10-24

**Authors:** Liyan Sun, Jingwen Bian, Yi Xin, Linqing Jiang, Linxuan Zheng

**Affiliations:** 1 College of Computer Science and Technology, Changchun University, Changchun City, Jilin Province, China; 2 School of Cultural and Media Studies, Changchun University of Science and Technology, Changchun City, Jilin Province, China; Government College University Faisalabad, PAKISTAN

## Abstract

Genome-wide association studies typically considers epistatic interactions as a crucial factor in exploring complex diseases. However, the current methods primarily concentrate on the detection of two-order epistatic interactions, with flaws in accuracy. In this work, we introduce a novel method called Epi-SSA, which can be better utilized to detect high-order epistatic interactions. Epi-SSA draws inspiration from the sparrow search algorithm and optimizes the population based on multiple objective functions in each iteration, in order to be able to more precisely identify epistatic interactions.

To evaluate its performance, we conducted a comprehensive comparison between Epi-SSA and seven other methods using five simulation datasets: DME 100, DNME 100, DME 1000, DNME 1000 and DNME3 100. The DME 100 dataset encompasses eight second-order epistasis disease models with marginal effects, each comprising 100 simulated data instances, featuring 100 SNPs per instance, alongside 800 case and 800 control samples. The DNME 100 encompasses eight second-order epistasis disease models without marginal effects and retains other properties consistent with DME 100. Experiments on the DME 100 and DNME 100 datasets were designed to evaluate the algorithms’ capacity to detect epistasis across varying disease models. The DME 1000 and DNME 1000 datasets extend the complexity with 1000 SNPs per simulated data instance, while retaining other properties consistent with DME 100 and DNME 100. These experiments aimed to gauge the algorithms’ adaptability in detecting epistasis as the number of SNPs in the data increases. The DNME3 100 dataset introduces a higher level of complexity with six third-order epistasis disease models, otherwise paralleling the structure of DNME 100, serving to test the algorithms’ proficiency in identifying higher-order epistasis. The highest average F-measures achieved by the seven other existing methods on the five datasets are 0.86, 0.86, 0.41, 0.56, and 0.79 respectively, while the average F-measures of Epi-SSA on the five datasets are 0.92, 0.97, 0.79, 0.86, and 0.97 respectively. The experimental results demonstrate that the Epi-SSA algorithm outperforms other methods in a variety of epistasis detection tasks. As the number of SNPs in the data set increases and the order of epistasis rises, the advantages of the Epi-SSA algorithm become increasingly pronounced.

In addition, we applied Epi-SSA to the analysis of the WTCCC dataset, uncovering numerous genes and gene pairs that might play a significant role in the pathogenesis of seven complex diseases. It is worthy of note that some of these genes have been relatedly reported in the Comparative Toxicogenomics Database (CTD). Epi-SSA is a potent tool for detecting epistatic interactions, which aids us in further comprehending the pathogenesis of common and complex diseases. The source code of Epi-SSA can be obtained at https://osf.io/6sqwj/.

## Introduction

Despite the significant progress made in identifying genes related to Mendelian genetic diseases, parsing the genetic basis of non-Mendelian (i.e., complex diseases) faces even more arduous challenges [1–4]. This challenge mainly stems from the phenomenon of epistasis, which significantly increases the complexity of genetic analysis. The prevailing view is that complex diseases are not caused by a single gene, but the result of the combined action of variations in multiple genes. These variations have a significant cumulative effect on the disease as a whole, although individually, their impact on the individual may be negligible. This cumulative effect is commonly referred to as epistatic interactions or multi-locus interactions [5–8].

With the rise of high-throughput genotyping and sequencing technologies, we are able to explore millions of single nucleotide polymorphisms (SNPs) at the individual level [9–12]. Genome-wide association studies (GWAS) as an emerging strategy has significantly promoted our understanding of the genetic basis of common and complex diseases. GWAS covers the genotyping analysis of hundreds of thousands of SNPs in thousands of individuals. In these studies, the detection of epistatic interactions provides a new perspective for disease genetics, thereby helping us to more fully understand these diseases and simultaneously providing new avenues for the prevention, diagnosis, and treatment of diseases [13–16].

In recent years, researchers have proposed a variety of strategies aimed at detecting epistatic interactions present in GWAS.

SNPHarvester [17] screens out SNP clusters significantly associated with the disease through multiple pathways and selects significant SNP clusters through rigorous statistical review. SNPRuler [18] is a method based on predictive rule reasoning to find epistatic interactions related to diseases, and it is the first method to ensure that it can find epistatic interactions without exhaustive search. BOOST [19] is a rapid method for detecting epistatic interactions. This method comprises two main steps: first, it employs a logical operation strategy to preliminarily screen pairs of SNPs; second, it carries out precise statistical analysis on the screened SNP pairs to evaluate their significant association with specific diseases. AntEpiSeeker [20] uses an innovative two-stage ant colony optimization algorithm to identify epistatic interactions within the framework of case-control studies. MACOED [21] as a multi-objective heuristic optimization method integrates logic regression and Bayesian network technology into the ant colony optimization algorithm to enhance the efficiency of the study. FHSA-SED [22] utilizes the Harmony Search Algorithm to identify 2-order epistasis in GWAS data. This method enhances the algorithm’s capacity to detect epistasis by combining both K2 and Gini as the objective functions for optimization. DECMDR [23] combines the differential evolution algorithm and classification-based multi-factor dimension reduction techniques, using CMDR as a fitness measure to explore potential epistatic interactions in GWAS. HS-MMGKG [24] combines harmony optimization algorithms and multiple optimization objectives and uses a novel strategy to combine the p-value and MDR method to increase the accuracy of the detection results. SEE [25] integrates eight evolutionary objectives and uses a new strategy based on sorting, exploration, and utilization to assess the association between SNP combinations and phenotypes. DL-GWAS [26] represents a cutting-edge deep-learning framework that employs convolutional neural networks (CNNs) to predict quantitative traits from SNPs in soybean genomes, eliminating the necessity for genotype imputation. This CNN-based model not only achieves higher accuracy but also demonstrates superior efficiency compared to conventional statistical approaches, providing a powerful tool for conducting genome-wide association studies. The multipopulation harmony search algorithm is specifically designed to identify high-order epistasis interactions [27]. This algorithm employs a multipopulation strategy to intensify the exploration of solution spaces, thereby effectively pinpointing intricate SNP interactions that are vital for comprehending the genetic foundation of diseases. Furthermore, it integrates a dynamic search mechanism that adjusts to the complexity of genetic data, rendering it a potent instrument for unearthing multiorder epistasis in biomedical research. SHEIB-AGM [28] is a random method based on an automatic gene matrix, in each iteration, it detects epistatic interactions on a higher-order SNP combination randomly according to the content of the gene matrix, and updates the gene matrix according to the detection results to ensure the detection capability of the algorithm. MP-HS-DHSI [27] is a multi-population Harmony Search Algorithm dedicated to the detection of high-order SNP interactions. It uses multiple criteria and multi-harmony memories to discover a set of candidate high-order SNP combinations associated with disease status. DeepCOMBI [29] utilizes CNNs within a deep-learning framework to predict phenotypes from SNPs in the context of GWAS. This innovative method not only achieves superior accuracy in phenotype prediction but also enhances the identification of genetic markers associated with complex traits, all without requiring genotype imputation. Furthermore, DeepCOMBI introduces an element of explainability to deep learning by employing layer-wise relevance propagation (LRP), which demystifies the decision-making processes of the CNNs. This transparent approach allows for the precise identification and selection of the most pertinent SNPs, which are then subjected to rigorous statistical testing, thereby enriching the discovery of significant genetic associations. BitEpi [30] is a fast and accurate method to test all possible combinations of up to four bi-allelic variants. It introduces a novel bitwise algorithm which is faster than established software and proposes a novel entropy statistic which is more accurate. EpiMOGA [31] is a multi-objective Genetic Algorithm for epistasis detection. It employs K2 and Gini to guide the search process of the genetic algorithm. A novel algorithm employs a multitasking framework that enhances the traditional Ant Colony Optimization by integrating a sophisticated pheromone update mechanism and local search heuristics, effectively navigating the intricate solution space to identify higher-order SNP interactions with precision [32]. This innovative method stands out for its ability to balance exploration and exploitation, ensuring a comprehensive and efficient search strategy. The paper proposes a novel hybrid algorithm that integrates membrane computing and harmony search for gene selection from expression and methylation data [33]. This unique method leverages the computational prowess of membrane computing in conjunction with the optimization potential of the harmony search algorithm, thereby facilitating the efficient identification of pertinent genes in bioinformatics analysis. SFMOABC [34] is a multi-objective Artificial Bee Colony Algorithm based on the scale-free network. It incorporates the scale-free network into the optimization to guide the update and selection of solutions. The Multitasking Harmony Search Algorithm-DHEI (MTHSA-DHEI) [35] is a sophisticated algorithm that utilizes a harmonious search framework specifically tailored for multitasking purposes. The primary objective of this algorithm is to proficiently navigate the solution space and pinpoint high-order Single Nucleotide Polymorphism (SNP) interactions. The paper proposes a novel method that innovatively employs a harmony search framework with explicit encoding to efficiently identify intricate genetic interactions [36]. This method distinguishes itself by its capacity to manage multiple tasks concurrently, thereby optimizing the search for high-order SNP interactions with precision. Although many algorithms for detecting epistatic interactions have emerged in recent years, they still have shortcomings in accuracy and efficiency. In this work, we proposes a new algorithm, Epi-SSA, which detects higher-order epistatic interactions in GWAS data through the Sparrow Search Algorithm (SSA) [37–40]. Compared to other existing algorithms, Epi-SSA exhibits the following significant advantages:

- Epi-SSA adopts an optimization strategy based on multiple objective functions, which can comprehensively evaluate SNP combinations related to the disease state, and enhances the depth of analysis of GWAS data.
- Epi-SSA can automatically identify the order of significant epistatic interactions related to the disease state without the need for users to preset the epistasis order, which is more in line with the actual needs of GWAS research.
- Epi-SSA generates new individuals based on the SNP weight vector in the iteration and updates the SNP weight vector in each iteration based on the detection results, guiding the evolution direction of the population.
- Epi-SSA solves the problem of false positives in detecting epistatic interactions based on a new strategy.

To verify the detection capability of Epi-SSA, we conducted comparative experiments between Epi-SSA and existing algorithms such as AntEpiSeeker, DECMDR, HS-MMGKG, SEE, SHEIB-AGM, SNPHarvester, and SNPRuler on five simulated datasets. These datasets cover 22 types of epistasis models and 3,800 simulated data files. The experimental results show that Epi-SSA demonstrates superior performance beyond other algorithms in the detection ability on the simulated dataset, especially in the identification of 3-order epistasis.

## Materials and methods

**Fig 1:**
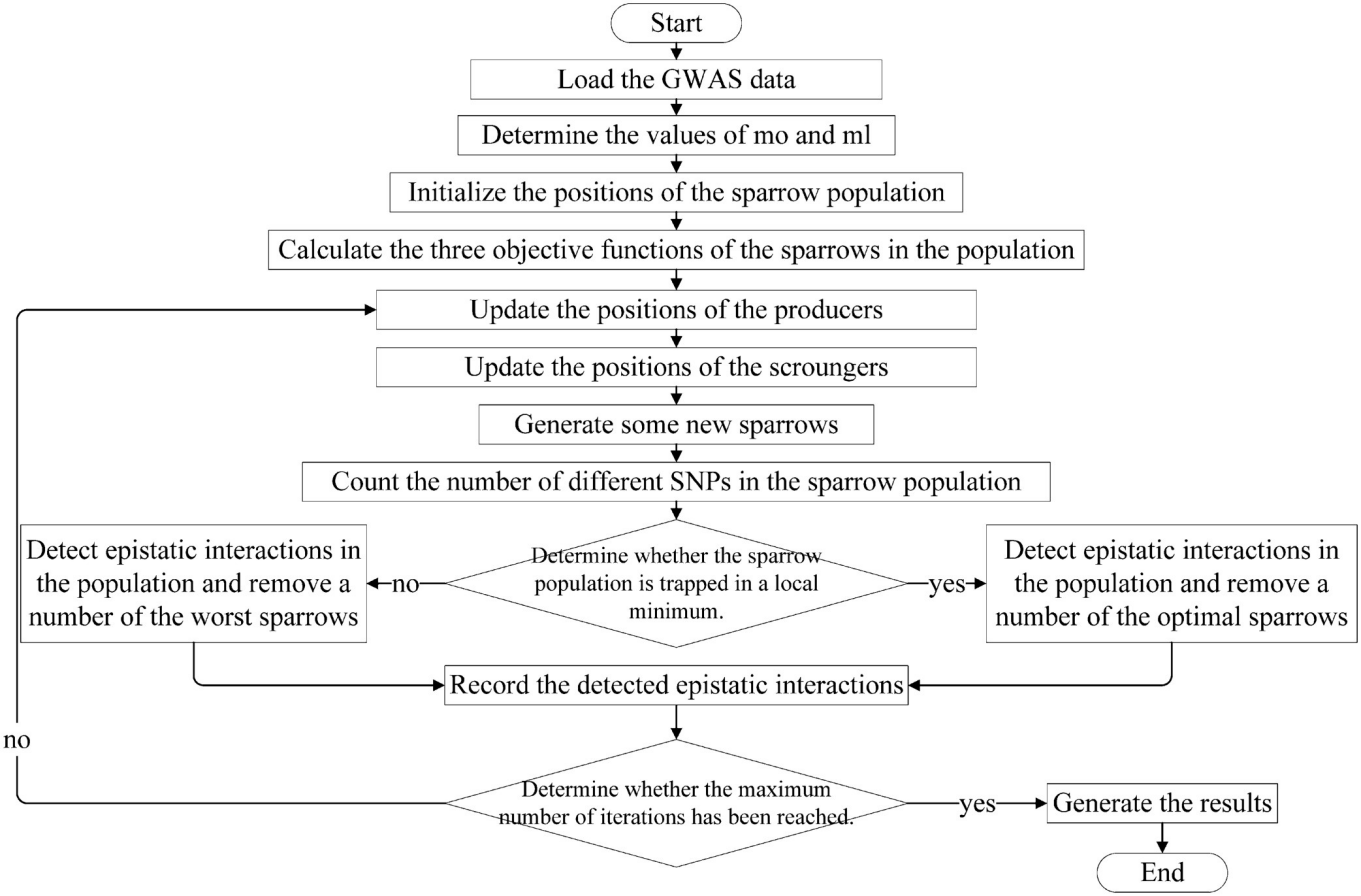
The overall structure and execution flow of Epi-SSA.

This work introduces a new algorithm Epi-SSA, which is based on the idea of the Sparrow Search Algorithm and focuses on identifying epistatic interactions related to diseases in GWAS data. This method guides the sparrow population to evolve in the direction of reducing the values of multiple objective functions through repeated iterative processes, and then obtains epistatic interactions related to diseases by detecting the partial optimal sparrows in each generation. The overall structure and execution flow of the algorithm are presented in Fig 1, with the subsequent sections of the text offering a meticulous breakdown of each procedural step.

### Determine the values of mo and ml

The Epi-SSA algorithm uses the maximum epistasis order (*mo*) [28] to define the upper limit of the order of epistatic interactions related to diseases that it can detect. The setting of this parameter can either be specified by the user according to the research requirements, or automatically calculated based on the number of samples in the GWAS data. The specific calculation method is shown in Eq (1). The purpose of setting mo is to limit the length when the evaluation function processes SNP combinations, and to ensure that throughout the entire operation process of the algorithm, the average number of samples for each genotype combination remains at the level of the natural constant *e*. This strategy effectively reduces the risk of the evaluation function possibly failing due to processing overly long SNP combinations, thereby enhancing the stability and reliability of the algorithm.

$$\begin{array}{c} mo=\lfloor \text{ln}\;(\text{min}(m0,m1))-0.5\rfloor \end{array}$$ (1)

Where *mo* represents the maximum epistasis order, *m*0 is the number of normal samples in the GWAS data, and *m*1 is the number of disease samples in the GWAS data.

The Epi-SSA algorithm restricts the scale of the contingency table generated during the calculation process of the evaluation function by setting the maximum length of the contingency table (ml). When using the evaluation function to analyze the correlation between SNP combinations and diseases, even for SNP combinations with the same length, due to the possible lack of samples for some genotype combinations, there is a difference in the actual length of the non-zero contingency table. This difference may lead to calculation deviations of the evaluation function on contingency tables of different lengths. Generally, the longer the length of the non-zero contingency table, the more significant the correlation between the SNP combination and the disease it reflects. In order to fairly assess this correlation, the Epi-SSA algorithm introduces a mechanism to control the length of the contingency table in the evaluation process. The setting of this parameter can either be specified by the user according to the research requirements, or automatically calculated based on the number of samples in the GWAS data. The specific calculation method is shown in Eq (2).

$$\begin{array}{c} ml=\lfloor \frac{\text{min}(m0,m1)}{10}\rfloor \end{array}$$ (2)

Where *ml* represents the maximum length of the contingency table, and the definitions of *m*0 and *m*1 are consistent with Eq (1).

### Initialize the positions of the sparrow population

Randomly generate *n* vectors with a length of *mo*, which represent the positions of *n* sparrows in the population. The position vector of each sparrow is defined according to Eq (3), which elaborately describes the composition of the position vector. During the iteration of the algorithm, the position vectors of the sparrows in the population will undergo a continuous optimization process, which aims to identify the gene epistatic interactions related to the disease.

$$\begin{array}{c} X_{i}=s_{i,1},s_{i,2},\ldots ,s_{i,j},\ldots ,s_{mo-1},s_{mo} \end{array}$$ (3)

Where *X*_*i*_ represents the position vector of the *i*-th sparrow in the population, and *i* ∈ [1, *n*]. Each element *s*_*i*,*j*_ in the vector *s*_*i*_ corresponds to the *s*_*i*,*j*_-th SNP in the GWAS dataset, and *s*_*i*,*j*_ ∈ [1, *N*], where *N* represents the total number of SNPs in the GWAS dataset.

### Calculate the three objective functions of the sparrows in the population

During the process of optimizing the population, the Epi-SSA algorithm adopts three objective functions to evaluate the position vector of each sparrow. These objective functions include K2, CE, and Gini, which are widely used when detecting epistatic interactions in GWAS data [27, 31, 34]. The detailed calculation methods of these functions are described in detail in Eq (4). They measure the correlation between the sparrow position vector and the disease from multiple dimensions. The lower the values of these objective functions, it indicates that the correlation between the corresponding position vector and the disease is more significant.

$$\begin{array}{cc} k2(X,Y)= & \prod_{x\in XG}\frac{(|YG|-1)!}{\left(m_{x}+|YG|-1\right)}\times \prod_{y\in YG}m_{xy}!\\ ce(X,Y)= & \sum_{x\in XG,y\in YG}p\left(x,y\right)\times \text{log}\;\frac{p(x,y)}{p(x)}\\ gini(X,Y)= & \sum_{x\in XG}\;p\left(x\right)\times \sum_{y\in YG}\;p\left(y|x\right)\times \left(1-p\left(y|x\right)\right) \end{array}$$ (4)

Where *X* is the vector of the sparrow’s position, and *Y* is the disease status of the sample. We use the K2 value (*k*2(*X*, *Y*)), the CE value (*ce*(*X*, *Y*)), and the Gini value (*gini*(*X*, *Y*)) to quantify the correlation between *X* and *Y*. *XG* represents the set of all possible combined genotypes corresponding to *X*. For instance, for a vector *X* with a length of 2, *XG* includes all possible genotype combinations, namely (0, 0), (0,1), (0,2), (1,0), (1,1), (1,2), (2,0), (2,1), (2,2). *YG* represents the set of sample states, and in the research of this work, it only comprises two values: 0 represents normal samples, and 1 represents disease samples. *m*_*x*_ is the number of samples in the data that have a specific combined genotype x on the SNPs corresponding to X, and *m*_*x*,*y*_ is the number of samples that have the combined genotype *x* and the sample state is *y*. *p*(*x*, *y*) is the ratio of *mx*, *y* to the total number of samples in the data, *p*(*x*) is the ratio of *m*_*x*_ to the total number of samples in the data, and *p*(*y*|*x*) is the ratio of *mx*, *y* to *m*_*x*_, which reflects the conditional probability that the sample state is *y* given a genotype *x*.

Although the three objective functions listed in Eq (4) are widely used in the algorithms for detecting epistatic interactions, they have a common limitation: these functions are calculated based on the contingency table between *X* and *Y*, and show significant sensitivity to the length of *XG*. Specifically, as the length of *XG* increases, the values of these three objective functions tend to decrease. To address this issue, Epi-SSA introduces an optimization strategy that is applicable to these three objective functions, which reduces the dependence on the length of *XG* by limiting the length of the contingency table to not exceed *ml*. The specific operation is as follows: Through the analysis of Eq (4), we can find that the value of each objective function is obtained through the cumulative summation of *XG*, and the smaller the value of the objective function, the stronger the correlation between *X* and *Y*. Therefore, during the calculation process, we sort the SNP combined genotypes on the contingency table and only retain the values of the smallest *ml* − 1 cells. For the remaining cells, we combine their samples to ensure that the maximum length of the contingency table does not exceed *ml*. This method effectively alleviates the bias of the objective function towards the length of *XG* and improves the accuracy and applicability of the algorithm.

**Fig 2:**
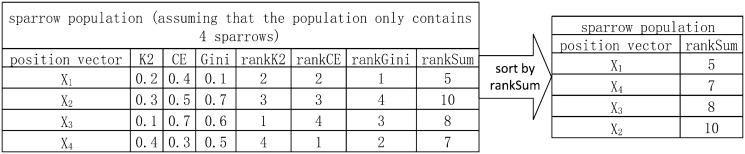
The strategy of sorting the sparrow population based on the rank.

In Epi-SSA, in order to effectively integrate the three objective functions as the optimization objectives of the population in the Sparrow Search Algorithm, we adopt a rank-based sorting strategy, akin to that utilized in SEE [25]. This process can be detailed through Fig 2. The specific steps are as follows: Firstly, we sort each sparrow in the population according to the independent values of each objective function, in order to determine the rankK2, rankCE, and rankGini values of each sparrow. Secondly, we accumulate the rankK2, rankCE, and rankGini values of each sparrow to obtain a comprehensive rank sum rankSum. Finally, we sort the sparrows in the population according to the rankSum value, arranged from small to large. According to the definitions of the three objective functions, the lower the rankSum value, it means that under the comprehensive consideration of these three objective functions, the correlation between the corresponding sparrow’s position vector and the disease state is more significant. Through this rank-based sorting method, Epi-SSA can efficiently identify and select the sparrows with a stronger correlation with the disease state within the multi-objective optimization framework, so as to optimize the performance of the algorithm.

### Update the positions of the producers

Based on the design concept of the Sparrow Search Algorithm, during the iteration, Epi-SSA divides the population into producers and scroungers, with the producers having a better fitness in the population compared to the scroungers. Alg 1 shows the process of updating the positions of the producers. Among them, the value range of the parameter *pd* is a decimal ranging from 0 to 1, representing the proportion of the producers in the population, which is 0.4 by default. The parameter *n* denotes the number of sparrows in the population. The parameter *st* is the safety threshold, and its value range is a decimal between 0.5 and 1, and the default value is 0.8. The parameter *mo* is the maximum epistasis order, which is specified by the user or obtained through calculation according to Eq (1).

**Algorithm 1**: Update the positions of the producers

**Input**: *pd*, *n*, *st*, *mo*.

**Output**: The position vectors of the sparrow population have been updated.

**1** **if** *rand(0,1)* < *st* **then**

**2**  **for** *i* ← 1 **to** *pd* × *n* **do**

**3**   Refer to the position vector of the *i*-th sparrow in the population as *X*_*i*_.

**4**   Randomly select one from all the SNPs in the GWAS data to replace a random position in *X*_*i*_, resulting in a new position vector of the sparrow, *newX*.

**5**   Add *newX* to the population.

**6**   Update the rankSum of the population and keep the population ordered.

**7**   **if** *the rankSum of newX* <*the rankSum of X*_*i*_ **then**

**8**    remove *X*_*i*_ from the population.

**9**   **else**

**10**    remove *newX* from the population.

**11**   **end**

**12**  **end**

**13** **else**

**14**  **for** *i* ← 1 **to** *pd* × *n* **do**

**15**   Refer to the position vector of the *i*-th sparrow in the population as *X*_*i*_. Randomly select $\frac{mo}{2}$ SNPs from the GWAS data to replace $\frac{mo}{2}$ random positions in *X*_*i*_ to generate a new sparrow position vector *newX*. Add *newX* to the population. Update the rankSum of the population and keep the population ordered. **if** *the rankSum of newX* <*the rankSum of X*_*i*_ **then**

**16**    remove *X*_*i*_ from the population.

**17**   **else**

**18**   **end**

**19**   remove *newX* from the population.

**20**  **end**

**21** **end**

### Update the positions of the scroungers

After the position vector of the producers has been updated, the Epi-SSA algorithm needs to update the scroungers in the population. The idea is for the better scroungers to move towards the producers, while the poorer scroungers move in a random direction, attempting to let the scroungers find a better position. The specific update method can refer to Alg 2, where the parameter *pss* vector records the probability of each SNP being selected in the GWAS data, and its dimension is consistent with the number of SNPs contained in the GWAS data. The Epi-SSA algorithm initializes the *pss* vector to an array comprised entirely of 1 in the startup phase. Along with the iterative process of the algorithm, the element values of the *pss* vector are correspondingly adjusted based on the results of the detected epistasis interactions in each iteration. The definition of the other parameters is the same as in Alg 1.

**Algorithm 2**: Update the positions of the scroungers

**Input**: *pd*, *n*, *st*, *mo*,*pss*.

**Output**: The position vectors of the sparrow population have been updated.

**1** **for** *i* ← *pd* × *n* + 1 **to** *n* **do**

**2**  **if** $i<\frac{n}{2}$ **then**

**3**   Refer to the position vector of the *i*-th sparrow in the population as *X*_*i*_.

**4**   Randomly select two producers in the population, and mark the producer with the lower rankSum as *P*.

**5**   Randomly select $\frac{mo}{2}$ SNPs from both *X*_*i*_ and *P* respectively to form a new vector with a length of mo and denoted as *newX*.

**6**   Add *newX* to the population.

**7**   Update the rankSum of the population and keep the population ordered.

**8**   **if** *the rankSum of newX* <*the rankSum of X*_*i*_ **then**

**9**    remove *X*_*i*_ from the population.

**10**   **else**

**11**    remove *newX* from the population.

**12**   **end**

**13**  **else**

**14**   From the GWAS data, randomly select *mo* SNPs according to the probability vector *pss* to form a new vector, denoted as *newX*.

**15**   Add *newX* to the population.

**16**   Update the rankSum of the population and keep the population ordered.

**17**   **if** *the rankSum of newX* <*the rankSum of X*_*i*_ **then**

**18**    remove *X*_*i*_ from the population.

**19**   **else**

**20**    remove *newX* from the population.

**21**   **end**

**22**  **end**

**23** **end**

### Generate *n* × *sd* new sparrows

After updating the position vector of the scroungers, the idea of the Sparrow Search Algorithm is that when the sparrows at the edge of the group perceive a threat, they will move towards the core area of the group; meanwhile, the sparrows in the center of the group will also conduct random exploration. In view of the characteristics of the GWAS data, the Epi-SSA algorithm simulates this behavior by generating some new sparrows. Specifically, the algorithm first randomly selects *n* × *sd* sparrows in the population. For each selected sparrow *a*, if it is the best sparrow in the current population, then the algorithm will randomly select *mo* SNPs from all the SNPs in the GWAS data according to the probabilities stored in the *pss* vector, form a new position vector (sparrow), and incorporate it into the population. If *a* is not the best sparrow, the algorithm will randomly select a producer b that is better than a, and then select half of the SNPs from the position vectors of both *a* and *b* to combine into a new position vector, and add this new vector (sparrow) to the population.

### Detect epistatic interactions in the population

The Epi-SSA algorithm employs a strategy that utilizes the K2 function to detect epistasis of order 2 to *mo* on mo-order SNP combinations. The core idea can be summarized as follows: Consider an SNP combination *X*, whose K2 value is calculated by Eq (4) and denoted as *k*2_*X*_. When an SNP *x* is removed from *X*, a new SNP combination *R* is formed, and its K2 value is denoted as *k*2_*R*_. If *x* is an SNP associated with the disease, or if *x* interacts with other SNPs in *X* to affect the disease (showing epistasis), then *k*2_*R*_ is greater than *k*2_*X*_; on the contrary, if *x* is noise, then *k*2_*R*_ should be less than or equal to *k*2_*X*_.

The Epi-SSA algorithm, based on this concept, takes the following steps to detect epistasis:

- Select *n* × *sd* optimal sparrows from the population (the top *n* × *sd* ranked in the population).
- For each sparrow position vector *X*, repeatedly attempt to remove all noise SNPs in *X* based on the K2 value.
- After the above process, a purified noise-free SNP combination *R* is obtained. If the length of *R* is greater than 1, use the G-test (according to Eq (5)) to assess the significance of the association between *R* and the disease.
- If the significance of the association between *R* and the disease is less than or equal to the user-defined threshold, record *R* as one of the results.
- To reduce the algorithm’s repeated focus on detected SNPs, in each iteration, update the weight *pss*[*x*] of each SNP *x* in *X*, with the formula *pss*[*x*] updated to *pss*[*x*] × 0.9.

$$\begin{array}{cc} F(X,Y)= & (|XG|-1)\times (|YG|-1).\\ E(x,y)= & m_{x}\times \frac{m_{y}}{m}.\\ g(X,Y)= & pvalue_{OfG}\left(2\times \sum_{x\in XG}\sum_{y\in YG}\;\text{ln}\;\frac{m_{x,y}}{E(x,y)}\right). \end{array}$$ (5)

Where *g*(*X*, *Y*) denotes the p-value obtained from the G-test for independence, which is used to evaluate the association between the SNP combination *X* and the phenotype *Y*. The significance of the relationship between *R* and the disease is assessed through *g*(*R*, *Y*). The variable *F*(*X*, *Y*) represents the degrees of freedom associated with the independence test. The count of samples with the SNP combination genotype *x* is denoted by *m*_*x*_, and the number of samples exhibiting phenotype *y* is given by *m*_*y*_. The total sample size is indicated by *m*, while *E*(*x*, *y*) is the expected count of samples with genotype *x* and phenotype *y*. The function *pvalue*_*OfG*_ calculates the p-value under the chi-square distribution, based on the statistical measures provided. The meanings of the remaining symbols are consistent with previous descriptions.

### Local optimum has been reached

The Epi-SSA algorithm assesses whether the search process has reached local optimum by analyzing the proportion of distinct SNPs in the population. The specific calculation method is shown in Eq (6). When the algorithm detects a local optimum, it will remove the top *n* × *sd* sparrows from the population; otherwise, it will remove the bottom *n* × *sd* sparrows.

$$\begin{array}{c} spasChaos=\frac{number\;of\;distinct\;SNPs}{n\times mo} \end{array}$$ (6)

Where *spasChaos* is utilized to assess whether the population has achieved the local optimum state. If *spasChaos* is less than the user-specified *thresholdSpasChaos* (with a default value of 0.6), Epi-SSA considers that the population has reached the local optimum state. *numSnps* refers to the total number of de-duplicated SNPs in the population, *n* represents the number of sparrows in the population, and *mo* indicates the maximum epistasis order.

### Generate the results

To reduce the false positives in the detection results, Epi-SSA proposes a new strategy to filter the results of epistasis detection, aiming to filter out the epistasis with relatively weaker association with the disease as noise. The specific steps are as follows:

- Sort all the epistasis in the results based on the significance of the G-test from strong to weak (ascending order of p-value).
- Assume that a total of *ne* epistatic interactions are detected. For each *i* ∈ [2, *ne*], calculate the ratio of the significance of the *i*th epistasis to the significance of the (*i* − 1)th epistasis, and record the *i* value corresponding to the largest ratio as *iBiggest*.
- Output the epistasis ranked before *iBiggest* in the results as the final detected epistasis to the result file, and ignore the epistasis ranked after *iBiggest* as noise.

By adopting this strategy, Epi-SSA greatly reduces the false positives in the detection results while maintaining the detection accuracy of the algorithm.

## Results and discussion

### Experiments on simulated datasets

To evaluate the capability of the Epi-SSA algorithm in the task of epistasis detection, this work carefully selected five simulated datasets to ensure a comprehensive assessment of the algorithm’s capabilities, the datasets can be obtained at https://osf.io/6sqwj/. The following is a detailed description of these datasets:

- DME 100 dataset: This dataset consists of 8 DME models, each model containing 100 GWAS simulated data files. Each file contains 100 SNPs, as well as 800 case and control samples. These models are derived from the DECMDR algorithm, and their penetrance tables can be found in S1 Table.
- DNME 100 Dataset: This dataset consists of 8 DNME models, each model also containing 100 GWAS simulated data files. Each file contains 100 SNPs, as well as 800 case and control samples. The DNME models were generated by the GAMETES [41] software, employing different minor allele frequency (MAF) value ranges [0.2, 0.4] and heritability value ranges [0.025, 0.05, 0.1, 0.2]. The relevant penetrance tables can be found in S2 Table.
- DME 1000 Dataset: This dataset is similar to the DME 100 dataset, with the only difference being that the number of SNPs contained in each GWAS data file has been increased to 1000.
- DNME 1000 Dataset: This dataset is similar to the DNME 100 dataset, with the only difference being that the number of SNPs in each GWAS data file has been increased to 1000.
- DNME3 100 Dataset: This dataset is composed of 8 DNME3 models, each model containing 100 GWAS simulated data files. Each file includes 100 SNPs, as well as 800 case and control samples. These models were generated by the GAMETES software, using different MAF value ranges [0.2, 0.4] and heritability value ranges [0.05, 0.1, 0.2]. The relevant penetrance tables can be found in S3 Table.

In this work, to evaluate the ability of different algorithms to detect epistasis on simulated datasets, we chose F-measure and Power as the metrics to measure the detection performance. These metrics are widely used when assessing the effectiveness of epistasis detection algorithms on simulated datasets [21, 24, 28, 31], and their calculation formulas are detailed in Equation Eq (7).

$$\begin{array}{cc} Recall= & \frac{TP}{TP+FN}\\ Precision= & \frac{TP}{TP+FP}\\ F-measure= & \frac{2}{\frac{1}{recall}+\frac{1}{precision}}\\ Power= & \frac{S}{100}. \end{array}$$ (7)

The higher the values of F-measure and Power, the better the algorithm performs in identifying epistatic interactions in the simulated dataset. When the algorithm performs epistasis detection on simulated GWAS data files and outputs results, *TP* represents the number of pathogenic epistatic interactions correctly detected. *FN* represents the number of pathogenic epistatic interactions that were not correctly detected. *FP* represents the number of SNP combinations unrelated to the disease that were incorrectly detected. The F-measure, as the harmonic mean of *recall* and *precision*, provides a quantitative measure of the overall performance of the algorithm. Specifically, the F-measure of the algorithm on a particular simulated model is determined by calculating the average F-measure of 100 simulated data files under that model. S represents the number of pathogenic epistasis SNP combinations accurately identified by the algorithm in 100 simulated data files.

In this work, we conducted an in-depth simulation experiment analysis of a series of algorithms, aiming to evaluate their ability to identify epistatic interactions. The algorithms involved include AntEpiSeeker, DECMDR, HS-MMGKG, SEE, SHEIB-AGM, SNPHarvester, SNPRuler, and Epi-SSA. Table 1 shows the parameters used by these algorithms on different simulated datasets in this paper. To ensure a fair comparison, the population size and the number of iterations are kept consistent when all algorithms are tested on the same dataset. Among them, AntEpiSeeker and SNPHarvester cannot detect 3-order epistasis, hence they cannot be executed on the DNME3 100 dataset.

**Table 1:** Algorithm parameter settings employed in the experiments on simulated data.

			parameter settings				
Algorithm	language	on all datasets	DME 100	DNME 100	DME 1000	DNME 1000	DNME3 100
AntEpiSeeker	C++	alpha = 1 iTopModel = 80 iTopLoci = 16 rou = 0.05 phe = 100 largehapsize = 6 smallhapsize = 3 pvalue = 0.05	iAntCount = 20 iItCountLarge = 25 iItCountSmall = 75 iEpiModel = 2	iAntCount = 20 iItCountLarge = 40 iItCountSmall = 120 iEpiModel = 2	iAntCount = 40 iItCountLarge = 1500 iItCountSmall = 4500 iEpiModel = 2	iAntCount = 40 iItCountLarge = 2000 iItCountSmall = 6000 iEpiModel = 2	Unable to execute
DECMDR	Java	s = 1 m = 0.5 r = 0.5	p = 20 g = 100 o = 2	p = 20 g = 160 o = 2	p = 40 g = 6000 o = 2	p = 40 g = 8000 o = 2	p = 20 g = 4000 o = 3
HS-MMGKG	Java	nsolution = 0 hmcr = 0.8 par = 0.4 fold = 5 pvalue = 0.05	hms = 20 tmax = 100 order = 2	hms = 20 tmax = 160 order = 2	hms = 40 tmax = 6000 order = 2	hms = 40 tmax = 8000 order = 2	hms = 20 tmax = 4000 order = 3
SEE	C++	pe = 0.4 stepInTable = 4 rn = 1 cG = 0.05	numPop = 20 maxIter = 100 order = 2	numPop = 20 maxIter = 160 order = 2	numPop = 40 maxIter = 6000 order = 2	numPop = 40 maxIter = 8000 order = 2	numPop = 20 maxIter = 4000 order = 3
SHEIB-AGM	Java	cG = 0.05 o = −1 pb = 0.8 cGc = 1	maxGen = 2000	maxGen = 3200	maxGen = 240000	maxGen = 320000	maxGen = 80000
SNPHarvester	Java	there is no parameter					Unable to execute
Epi-SSA	Java	pd = 0.4 sd = 0.2 st = 0.8 ml = 0 mo = 0 seed = 0 cG = 0.05 thresholdSpasChaos = 0.6	n = 100 maxG = 20	n = 20 maxG = 160	n = 40 maxG = 6000	n = 40 maxG = 8000	n = 20 maxG = 4000

**Fig 3:**
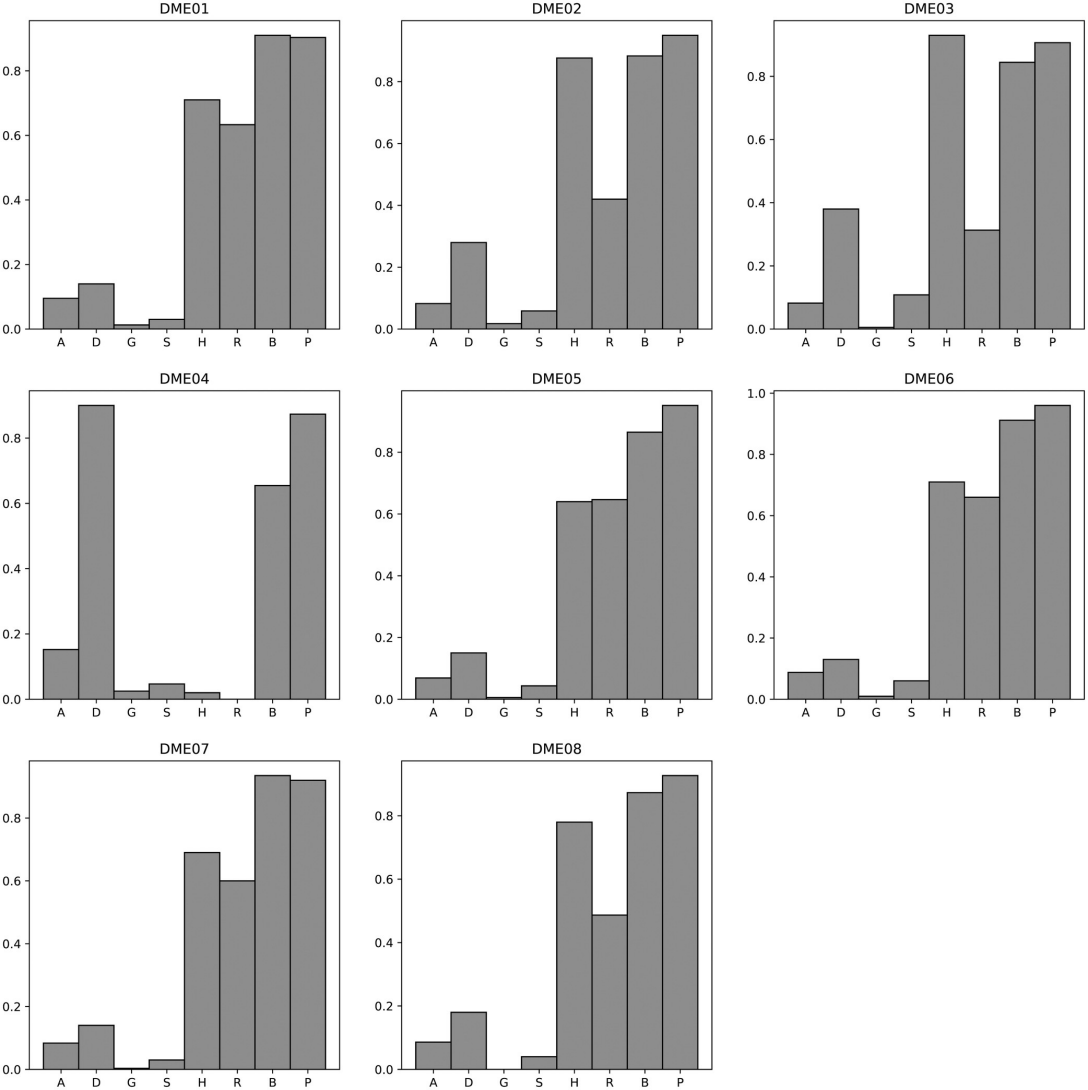
F-measure comparisons between AntEpiSeeker(A), DECMDR(D), HS-MMGKG(G), SEE(S), SHEIB-AGM(B), SNPHarvester(H), SNPRuler(R) and Epi-SSA(P) on the DME 100 dataset.

Fig 3 displays a comparative analysis of the F-measure of different algorithms on the DME 100 dataset. More detailed results can be found in S4 Table. The average F-measure and standard deviation of these algorithms on the DME 100 dataset are as follows: AntEpiSeeker (0.09, 0.03), DECMDR (0.29, 0.26), HS-MMGKKG (0.01, 0.01), SEE (0.05, 0.03), SHEIB-AGM (0.86, 0.09), SNPHarvester (0.67, 0.28), SNPRuler (0.47, 0.23), and Epi-SSA (0.92, 0.03). For the comparison results of Power and Execution time on the DME 100 dataset, please refer to S5 and S6 Tables, S1 and S2 Figs. The average Power and standard deviation of these algorithms on the DME 100 dataset are as follows: AntEpiSeeker (0.72, 0.11), DECMDR (0.29, 0.26), HS-MMGKKG (0.07, 0.08), SEE (0.08, 0.07), SHEIB-AGM (0.99, 0.03), SNPHarvester (0.67, 0.28), SNPRuler (0.71, 0.34), and Epi-SSA (0.94, 0.03). The execution time of these algorithms on the DME 100 dataset are shown in S6 Table and S2 Fig. The experimental results clearly indicate that the Epi-SSA algorithm outperforms other algorithms in identifying epistatic interactions on the DME 100 dataset. Although slightly lower than the SHEIB-AGM algorithm in terms of Power, Epi-SSA shows a better performance in F-measure, which is attributed to its effectiveness in reducing false positives in the detection results.

**Fig 4:**
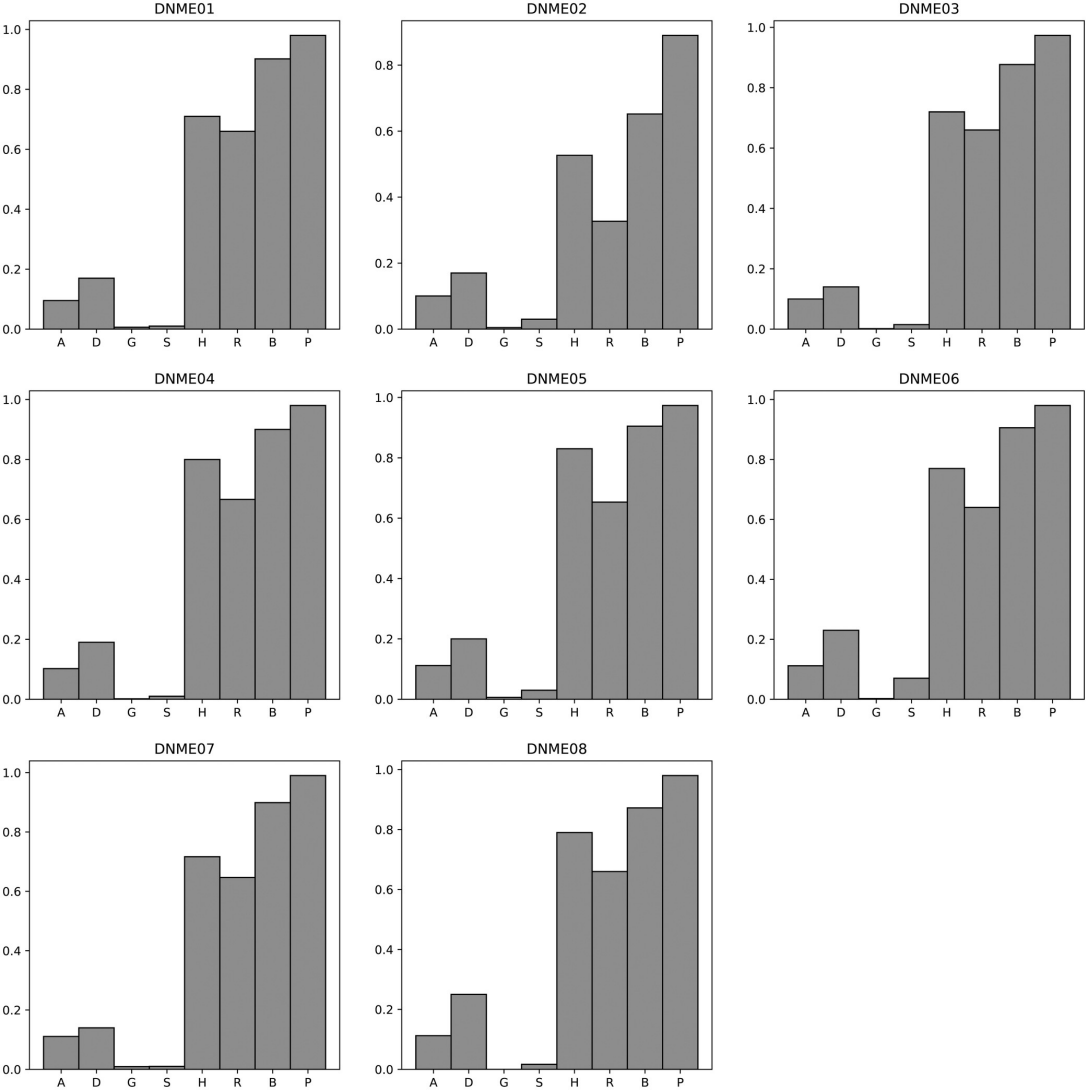
F-measure comparisons between AntEpiSeeker(A), DECMDR(D), HS-MMGKG(G), SEE(S), SHEIB-AGM(B), SNPHarvester(H), SNPRuler(R) and Epi-SSA(P) on the DNME 100 dataset.

Fig 4 presents a comparative analysis of the F-measure for various algorithms when applied to the DNME 100 dataset. For an exhaustive view of the results, please refer to S7 Table. The mean F-measure and corresponding standard deviation for each algorithm on the DNME 100 dataset are detailed below: AntEpiSeeker (0.11, 0.01), DECMDR (0.19, 0.04), HS-MMGKKG (0.004, 0.00), SEE (0.02, 0.02), SHEIB-AGM (0.86, 0.09), SNPHarvester (0.73, 0.09), SNPRuler (0.61, 0.12), and Epi-SSA (0.97, 0.03). For a detailed examination of the Power and Execution time comparison on the DNME 100 dataset, S8 and S9 Tables, as well as S3 and S4 Figs, should be consulted. The mean Power values for these algorithms on the DNME 100 dataset are as follows: AntEpiSeeker (0.88, 0.05), DECMDR (0.19, 0.04), HS-MMGKKG (0.02, 0.02), SEE (0.03, 0.02), SHEIB-AGM (0.96, 0.09), SNPHarvester (0.73, 0.17), SNPRuler (0.92, 0.10), and Epi-SSA (0.98, 0.03). The execution times for these algorithms on the DNME 100 dataset are delineated in S9 Table and S4 Fig. The experimental results unequivocally demonstrate the superiority of the Epi-SSA algorithm in detecting epistatic interactions within the DNME 100 dataset. Consistently, across various model datasets, Epi-SSA exhibits a pronounced advantage in the detection capability of epistatic interactions.

**Fig 5:**
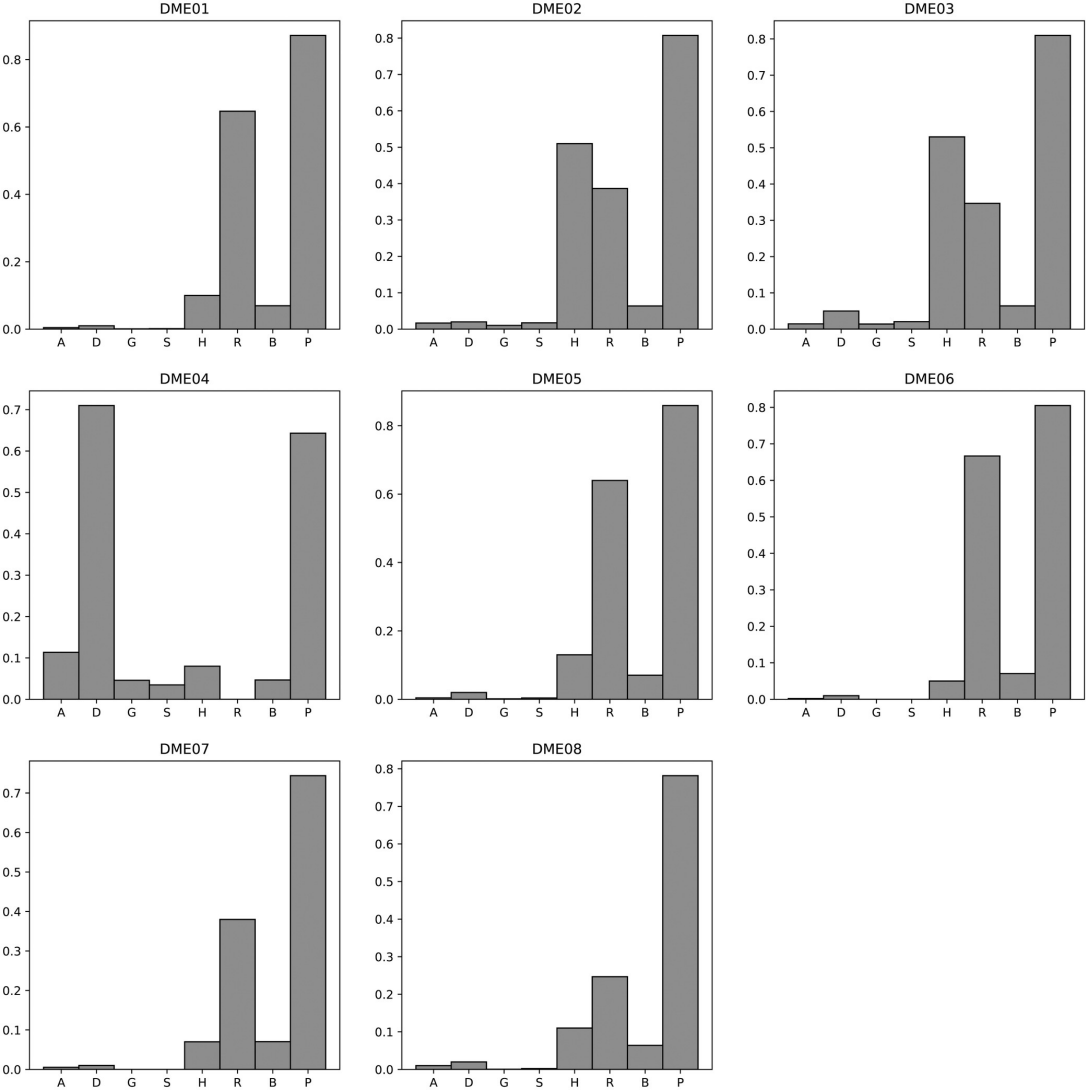
F-measure comparisons between AntEpiSeeker(A), DECMDR(D), HS-MMGKG(G), SEE(S), SHEIB-AGM(B), SNPHarvester(H), SNPRuler(R) and Epi-SSA(P) on the DME 1000 dataset.

Fig 5 presents a comparative analysis of the F-measure for an array of algorithms applied to the DME 1000 dataset. For an in-depth examination of the results, S10 Table provides further details. The average F-measure and standard deviation across these algorithms on the DME 1000 dataset are detailed as follows: AntEpiSeeker (0.02, 0.04), DECMDR (0.11, 0.24), HS-MMGKKG (0.01, 0.02), SEE (0.01, 0.01), SHEIB-AGM (0.06, 0.01), SNPHarvester (0.20, 0.20), SNPRuler (0.41, 0.23), and Epi-SSA (0.79, 0.07). For a comprehensive comparison of Power and Execution time on the DME 1000 dataset, S11 and S12 Tables, as well as S5 and S6 Figs, should be consulted. The mean Power and standard deviation for these algorithms on the DME 1000 dataset are as follows: AntEpiSeeker (0.20, 0.30), DECMDR (0.11, 0.24), HS-MMGKKG (0.19, 0.32), SEE (0.10, 0.18), SHEIB-AGM (0.99, 0.02), SNPHarvester (0.20, 0.20), SNPRuler (0.62, 0.35), and Epi-SSA (0.90, 0.05). The execution times for these algorithms on the DME 1000 dataset are delineated in S12 Table and S6 Fig. The experimental data conclusively demonstrate the superiority of the Epi-SSA algorithm in identifying epistatic interactions within the DME 1000 dataset. Notably, even with an increase in the number of SNPs to 1000, Epi-SSA sustains its remarkable capacity for detecting epistatic interactions. When juxtaposed with the SHEIB-AGM algorithm, Epi-SSA achieves a significant reduction in the false positive rate within the detection outcomes.

**Fig 6:**
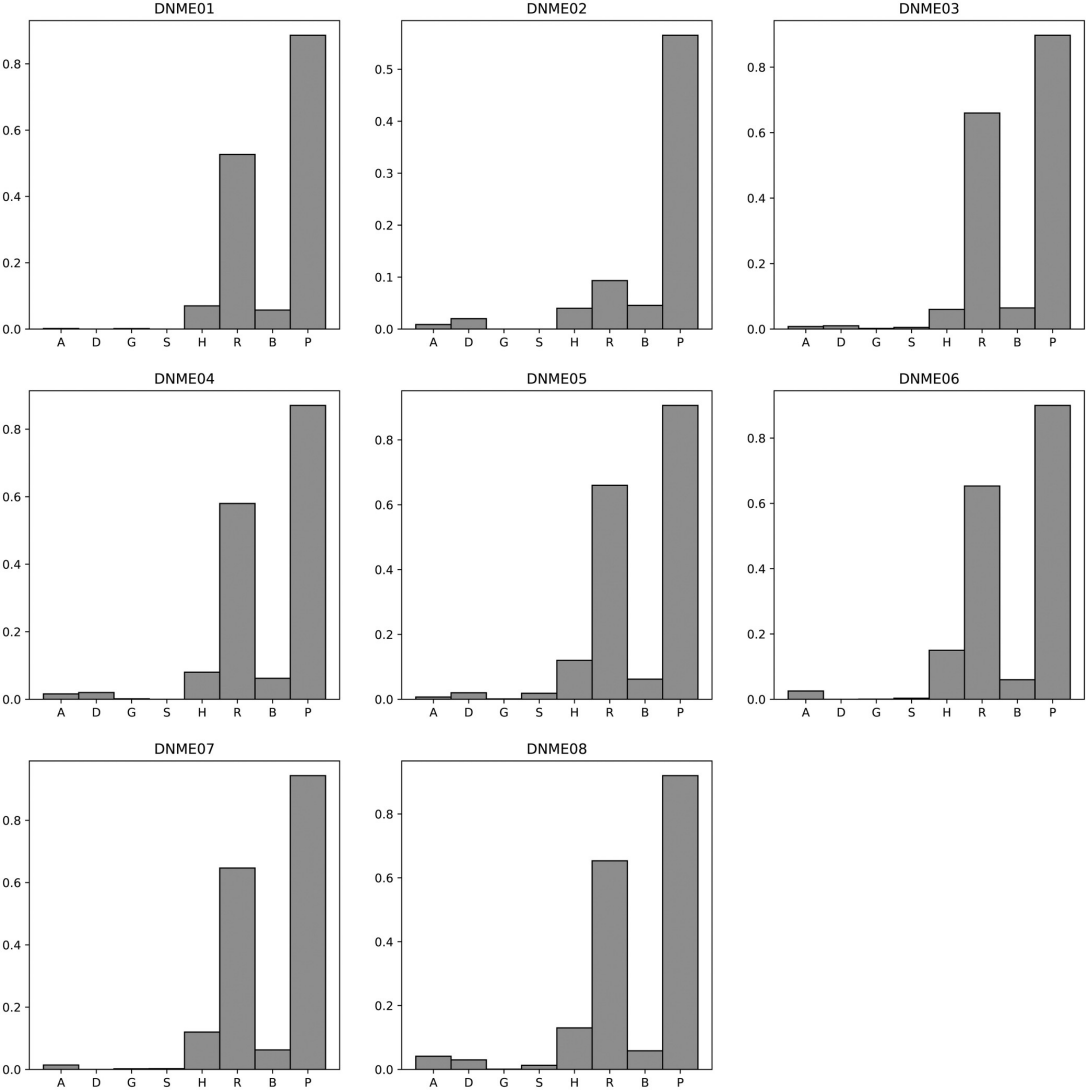
F-measure comparisons between AntEpiSeeker(A), DECMDR(D), HS-MMGKG(G), SEE(S), SHEIB-AGM(B), SNPHarvester(H), SNPRuler(R) and Epi-SSA(P) on the DNME 1000 dataset.

Fig 6 illustrates a comparative analysis of the F-measure for various algorithms when evaluated on the DNME 1000 dataset. For a more granular examination of the outcomes, S13 Table offers an extensive breakdown. The mean F-measure and standard deviation for these algorithms on the DNME 1000 dataset are presented as follows: AntEpiSeeker (0.02, 0.01), DECMDR (0.01, 0.01), HS-MMGKKG (0.001, 0.00), SEE (0.01, 0.01), SHEIB-AGM (0.06, 0.01), SNPHarvester (0.10, 0.04), SNPRuler (0.56, 0.19), and Epi-SSA (0.86, 0.12). Further insights into the Power and execution time of these algorithms on the DNME 1000 dataset are detailed in S14 and S15 Tables, as well as S7 and S8 Figs. The mean Power and standard deviation for the algorithms on the DNME 1000 dataset are as follows: AntEpiSeeker (0.18, 0.15), DECMDR (0.01, 0.01), HS-MMGKKG (0.02, 0.01), SEE (0.02, 0.02), SHEIB-AGM (0.97, 0.08), SNPHarvester (0.10, 0.04), SNPRuler (0.84, 0.29), and Epi-SSA (0.95, 0.12). The experimental results conclusively demonstrate that the Epi-SSA algorithm excels in identifying epistatic interactions within the DNME 1000 dataset, showcasing its superior performance over other competing algorithms.

**Fig 7:**
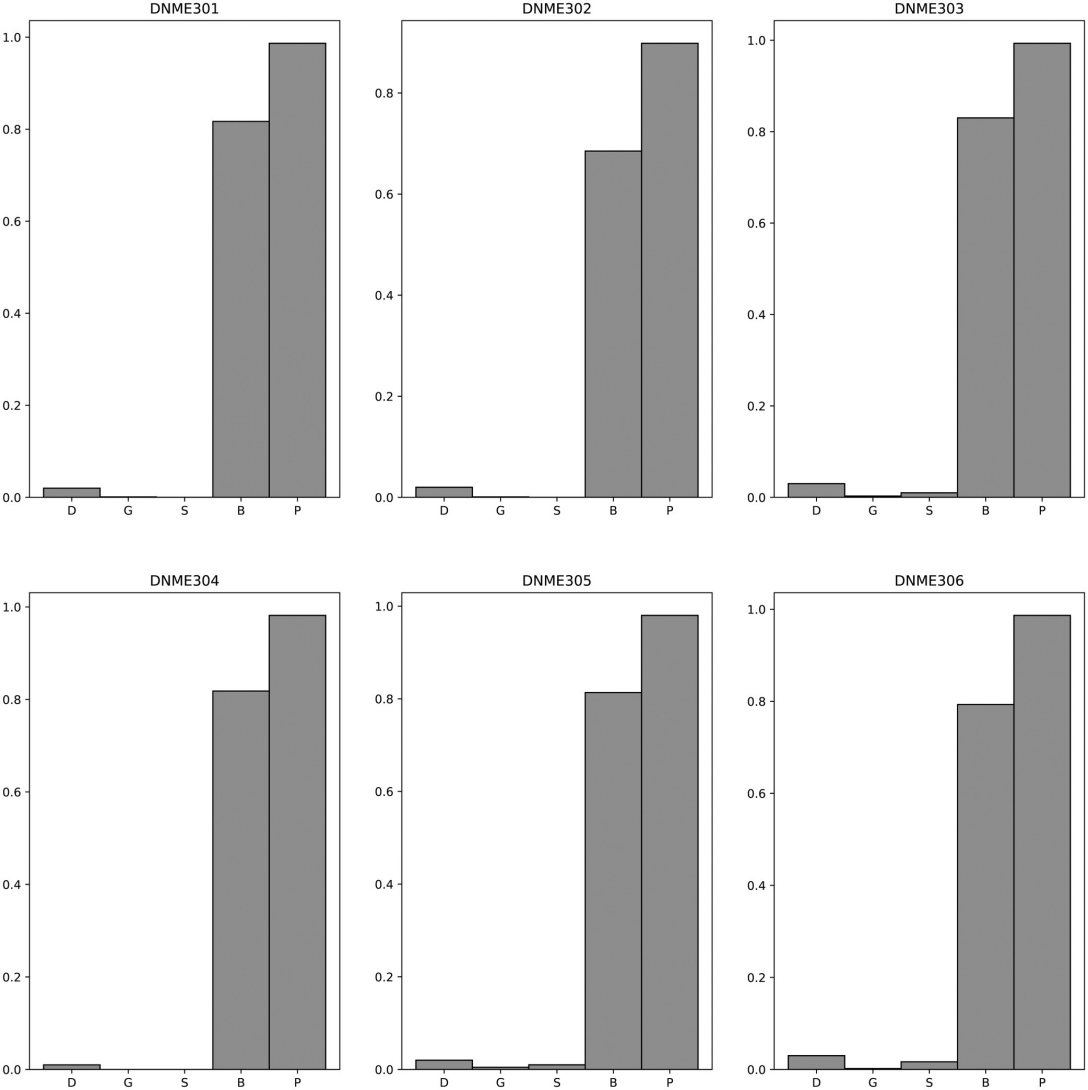
F-measure comparisons between DECMDR(D), HS-MMGKG(G), SEE(S), SHEIB-AGM(B) and Epi-SSA(P) on the DNME3 100 dataset.

Fig 7 presents a comparative analysis of the F-measure for various algorithms when applied to the DNME3 100 dataset. Comprehensive results are detailed in S16 Table. The mean F-measure and standard deviation for these algorithms on the DNME3 100 dataset are as detailed below: DECMDR (0.02, 0.01), HS-MMGKKG (0.002, 0.00), SEE (0.01, 0.01), SHEIB-AGM (0.79, 0.05), and Epi-SSA (0.97, 0.04). For an in-depth comparison of Power and execution time on the DNME3 100 dataset, refer to S17 and S18 Tables, as well as S9 and S10 Figs. The mean Power and standard deviation for these algorithms on the DNME3 100 dataset are as follows: DECMDR (0.02, 0.01), HS-MMGKKG (0.02, 0.02), SEE (0.01, 0.01), SHEIB-AGM (0.99, 0.02), and Epi-SSA (0.95, 0.04). The experimental results provide a clear indication that the Epi-SSA algorithm holds a significant advantage over other algorithms in identifying epistatic interactions within the DNME3 100 dataset. This advantage is particularly pronounced when detecting 3-order epistatic interactions, where the Epi-SSA algorithm consistently exhibits its exceptional performance.

### Experiments on real data

We obtained a real dataset from the Wellcome Trust Case Control Consortium (WTCCC) [42], which covers about 14,000 diseased samples for seven common complex diseases: Bipolar Disorder, Coronary Artery Disease, Crohn’s Disease, Hypertension, Rheumatoid Arthritis, Type 1 Diabetes, and Type 2 Diabetes. The dataset is not publicly available. Access can be requested from the owners at https://www.wtccc.org.uk/info/access_to_data_samples.html and https://www.sanger.ac.uk/legal/DAA/MasterController. In addition, the dataset also includes a shared control group of about 3,000 samples. For a detailed description of the dataset, you can refer to S19 Table. We combined the cases with the shared control group for each disease to construct seven GWAS data. In further analysis, following the recommendations of the WTCCC, we excluded samples and SNPs that needed to be removed, as well as those SNPs that did not show variation in all samples. After these filtering steps, we obtained the seven cleaned GWAS data presented in Table 2.

**Table 2:** The real GWAS data for the seven common complex diseases.

data	disease name	number of SNPs	number of cases	number of controls	number of samples
bd_gwas	Bipolar Disorder	458922	1868	2938	4806
cad_gwas	Coronary Artery Disease	458743	1926	2938	4864
cd_gwas	Crohn’s Disease	459472	1748	2938	4686
ht_gwas	Hypertension	458851	1952	2938	4890
ra_gwas	Rheumatoid Arthritis	458854	1860	2938	4798
t1d_gwas	Type 1 Diabetes	459244	1963	2938	4901
t2d_gwas	Type 2 Diabetes	459112	1924	2938	4862

In this work, we applied the Epi-SSA algorithm to analyze the seven GWAS data listed in Table 2 in order to identify epistatic interactions associated with the seven common complex diseases. We detected a large number of epistatic interactions, with some of the results shown in Table 3. Specifically, we found 5,264 epistatic interactions in Bipolar Disorder, 628,817 in Coronary Artery Disease, 3,978 in Crohn’s Disease, 10,013 in Hypertension, 66,642 in Rheumatoid Arthritis, 104,743 in Type 1 Diabetes and 6,334 in Type 2 Diabetes. A detailed list of all detected results has been provided in S20 Table.

**Table 3:** Part of epistatic interactions found by Epi-SSA (n = 600 maxG = 800000).

p-value	SNP1	SNP2	SNP3	SNP4	SNP5	SNP6
**Bipolar Disorder**						
0	rs6599159	rs3845903	rs9368536	rs1909936	rs16909286	
0	rs2023974	rs1024592	rs1909936	rs1556811		
0	rs6923059	rs1909936	rs797493	rs396395		
0	rs1553460	rs10461624	rs1925454	rs1094138		
0	rs1553460	rs41478747	rs7718172	rs41323346		
0	rs6852266	rs1553460	rs16892342	rs17144728		
**Coronary Artery Disease**						
0	rs1541658	rs9804878	rs16957197	rs4149696	rs6643336	rs979357
0	rs6531531	rs793014	rs10074255	rs17063729	rs16926588	rs5928104
0	rs6848027	rs6531531	rs4868979	rs16938648	rs16969155	rs1199460
0	rs6836401	rs41511044	rs159171	rs6059136	rs16981516	rs41369746
0	rs6777905	rs13126272	rs4881411	rs7189731	rs3269	rs1481162
0	rs7653441	rs17129333	rs9288782	rs11921179	rs41478844	rs4799934
**Crohn’s Disease**						
0	rs16869934	rs16888603	rs494483	rs17083420	rs2332903	rs9956765
0	rs6816863	rs6871834	rs494483	rs16895349	rs7787285	
0	rs1398832	rs7091562	rs4471699	rs13339951	rs17002802	
0	rs1933641	rs1398832	rs7213498	rs10426571	rs17004382	
0	rs16825583	rs16856907	rs1553460	rs6460236	rs16906441	
0	rs17577123	rs494483	rs16878847	rs302925		
**Hypertension**						
0	rs4867173	rs11050927	rs10843660	rs17078208	rs13332100	
0	rs1553460	rs4867173	rs11244965	rs359366	rs9927288	
0	rs17046143	rs6840033	rs17116117	rs7124582	rs3764220	
0	rs345265	rs7628932	rs16837871	rs4867173	rs7720671	
0	rs12060579	rs825148	rs2098536	rs1432960	rs4867173	
0	rs17465032	rs2766987	rs17116117	rs2373907		
**Rheumatoid Arthritis**						
0	rs2298296	rs1369036	rs16863294	rs17032985	rs3129768	rs851236
0	rs41522846	rs3889096	rs692016	rs7176759	rs16942813	
0	rs2490225	rs532806	rs507415	rs17104722	rs4829106	
0	rs9268402	rs3129934	rs16872017	rs2001097	rs16970572	
0	rs2001099	rs6962909	rs41454849	rs2652020	rs5943990	
0	rs3135376	rs2395167	rs16907620	rs17104722	rs5909232	
**Type 1 Diabetes**						
0	rs6748474	rs10515517	rs3094123	rs2857212	rs4350455	rs6113065
0	rs644045	rs9261376	rs3129933	rs11985334	rs2468600	
0	rs17141406	rs2074508	rs1494160	rs2071278	rs692143	
0	rs408359	rs9268429	rs9261389	rs2170416	rs11911295	
0	rs3177928	rs4144562	rs2517591	rs2395161	rs16925381	
0	rs17495612	rs3177928	rs11158	rs2111428	rs931770	
**Type 2 Diabetes**						
0	rs1324132	rs6921387	rs17102342	rs6561351	rs7333888	
0	rs16891175	rs9480510	rs1324132	rs1477523	rs711295	
0	rs16837871	rs16998188	rs1324132	rs10092007	rs505063	
0	rs1447910	rs12198368	rs1324132	rs6938374	rs663335	
0	rs2062567	rs41441244	rs1590392	rs1324132	rs468453	
0	rs17036088	rs1448952	rs16870674	rs1324132	rs5951720	

**Fig 8:**
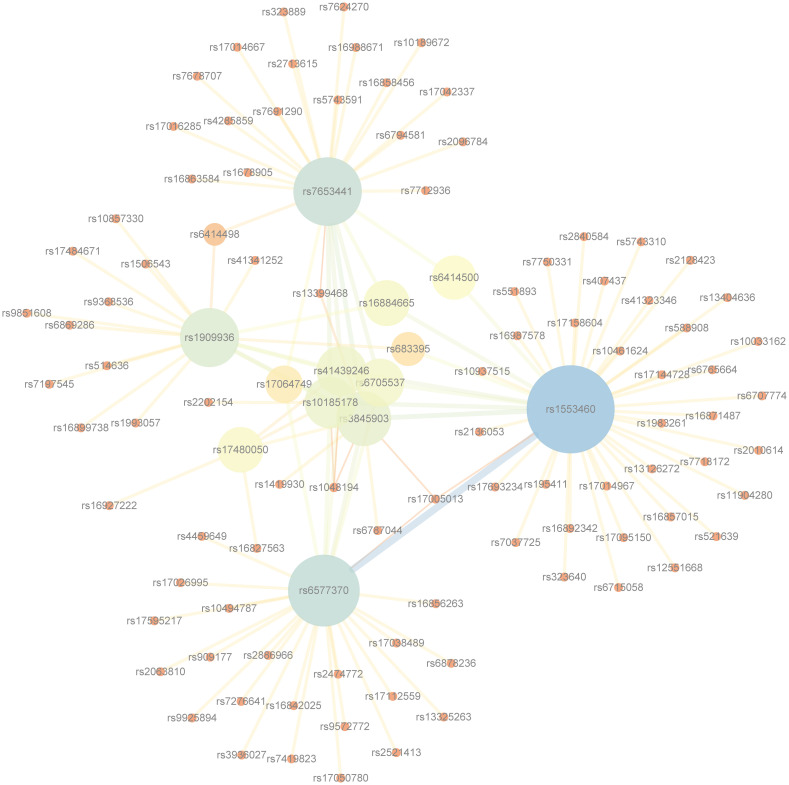
The SNP network of the epistatic interactions detected for Bipolar Disorder.

Fig 8 displays the SNP network drawn based on the detection results of Bipolar Disorder using Cytoscape software. For the readability of the network, only SNP pairs with occurrences not less than 4 in the results were included. As shown in the figure, SNPs such as rs7653441, rs1909936, rs1553460, and rs6577370 are of significant importance for the study of Bipolar Disorder. The SNP networks for the other six diseases can be referred to S11–S16 Figs.

The SNPs from the detection results were mapped to the relevant genes through the dbSNP database [43, 44]. For each common and complex disease studied, we statistically analyzed the frequency of occurrence of the genes and gene pairs in the detection results. Genes and gene pairs with a higher frequency of occurrence may play a key role in the occurrence and development of related diseases. To further explore the biological significance of these genes, we conducted an in-depth search using the CTD database (the Comparative Toxicogenomics Database) [45]. In the records of the CTD database, DE represents genes with direct evidence supporting their association with specific diseases, NDE refers to genes that are associated with diseases but lack direct evidence, and NF indicates genes for which there are currently no records showing a direct connection with diseases. For the statistics of genes pairs and gene in the detection results, some results are displayed in Tables 4 and 5, while the complete data are included in S21 and S22 Tables.

**Table 4:** Part of gene pairs of the epistastic interactions detected on the seven GWAS data using Epi-SSA.

**Bipolar Disorder**				
FNDC3B	NDE	LRIG1	NDE	316
H3C11	NDE	H4C13	NDE	15
CENPN	NDE	MYO3B	NDE	8
ACSL1	NDE	ACSL1	NDE	6
A2M	NDE	KLRG1	NDE	6
CSGALNACT1	NDE	GRIN2A	DE	2
**Coronary Artery Disease**				
AIFM1	NDE	RAB33A	NDE	678
PHEX	NDE	PTCHD1-AS	NDE	603
FNDC3B	NDE	MRAS	DE	156
ACSL1	NDE	GUCY1A1	DE	12
ESR1	DE	FRMPD4	NDE	6
DMD	NDE	LDB2	NDE	5
**Crohn’s Disease**				
ATG16L1	DE	LDB2	NDE	18
TBC1D32	NDE	WWC1	NDE	10
IRGM	DE	PTGFRN	NDE	8
IL23R	DE	KCNIP4	NDE	5
CD274	NDE	RRP15	NDE	4
BTG3	NDE	CXADR	NDE	4
**Hypertension**				
SCOC	NDE	SCOC-AS1	NDE	144
CTNNA3	NDE	LRRTM3	NDE	6
CTC-338M12.4	NDE	TRIM52	NDE	5
BCL9L	NDE	CXCR5	NDE	4
HTR3B	NDE	PDE3A	DE	2
CHRM2	NDE	EDN1	DE	2
**Rheumatoid Arthritis**				
BTNL2	NDE	HLA-DPA1	NDE	109
HLA-DQA2	DE	TSBP1	NDE	101
CFB	NDE	NELFE	NDE	52
PON1	DE	TAP2	NDE	5
HLA-DRA	NDE	PTPN22	DE	4
HLA-DPB1	DE	IKZF3	DE	2
**Type 1 Diabetes**				
LY6G6C	NDE	MPIG6B	NDE	760
CDSN	NDE	PSORS1C1	NDE	319
HLA-DQA1	DE	HLA-DQA2	NDE	95
AGPAT1	NDE	TNF	DE	26
GLIS3	DE	TSBP1	NDE	8
BACH2	DE	HCG20	NDE	6
**Type 2 Diabetes**				
CLIC5	NDE	LRIG1	NDE	18
ACSL1	NDE	ACSL1	NDE	16
SBF2	NDE	TCF7L2	DE	3
CSGALNACT1	NDE	GLIS3	DE	3
GCLC	DE	KIAA1671	NDE	2
HTR3B	NDE	ITGA1	DE	2

**Table 5:** Part of genes of the epistastic interactions detected on the seven GWAS data using Epi-SSA.

**Bipolar Disorder**		
FNDC3B	NDE	1154
LRIG1	NDE	992
CSGALNACT1	NDE	439
NTNG1	DE	4
GRIN2A	DE	3
PDE4B	DE	2
**Coronary Artery Disease**		
FNDC3B	NDE	56444
ACSL1	NDE	28744
DMD	NDE	17273
MRAS	DE	246
PHACTR1	DE	144
LDB2	NDE	90
**Crohn’s Disease**		
KCNIP4	NDE	799
RRP15	NDE	532
LDB2	NDE	492
ATG16L1	DE	99
IL23R	DE	30
IRGM	DE	28
**Hypertension**		
CHRM2	NDE	871
HTR3B	NDE	694
GAN	NDE	243
TGFA	DE	6
EDN1	DE	5
STK39	DE	5
**Rheumatoid Arthritis**		
TSBP1	NDE	9204
BTNL2	NDE	4080
TAP2	NDE	2298
HLA-DPB1	DE	1613
HLA-DQA2	DE	818
PTPN22	DE	8
**Type 1 Diabetes**		
HLA-DQA2	NDE	13335
TSBP1	NDE	10129
AGPAT1	NDE	4716
HLA-DQA1	DE	1519
TNF	DE	71
GLIS3	DE	57
**Type 2 Diabetes**		
CLIC5	NDE	1025
HTR3B	NDE	614
CSGALNACT1	NDE	404
TCF7L2	DE	7
PPARGC1A	DE	6
GLIS3	DE	6

**Fig 9:**
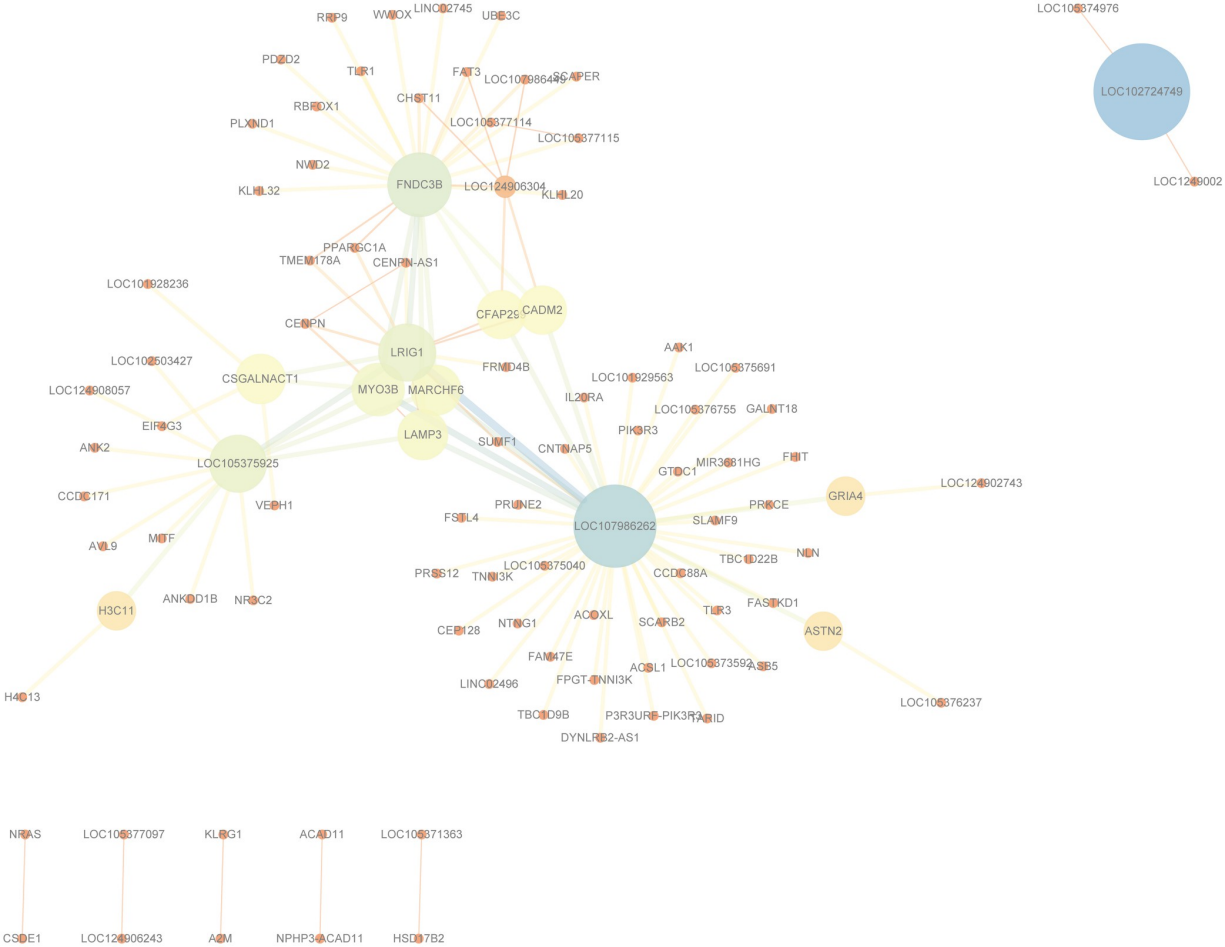
The gene network of the epistatic interactions detected for Bipolar Disorder.

Fig 9 displays the gene network drawn based on the detection results of Bipolar Disorder using Cytoscape software [46]. For the readability of the network, only gene pairs with occurrences not less than 4 in the results were included. As shown in the figure, genes such as FNDC3B, LOC107986262, LRIG1, and LOC105375925 are of significant importance for the study of Bipolar Disorder. The gene networks for the other six diseases can be referred to S17–S22 Figs.

## Conclusion

In this work, we introduce a novel method for detecting epistatic interactions in GWAS data, termed Epi-SSA. This method is designed based on a multi-objective Sparrow Search Algorithm. To evaluate the performance of the Epi-SSA algorithm, extensive experiments were conducted on five simulated datasets generated using GAMETES_2.1. These experiments compared its capabilities in detecting epistasis from various perspectives with other algorithms.

Initially, the experimental results on the DME 100 and DNME 100 datasets demonstrated that the Epi-SSA algorithm has superior detection capabilities when faced with multiple potential pathogenic models. Although occasionally slightly lower in power compared to the SHEIB-AGM algorithm, Epi-SSA significantly reduced the number of false positives in the results. Subsequently, the results from the DME 1000 and DNME 1000 datasets indicated that as the number of SNPs in the GWAS data increases, the detection capability of the Epi-SSA algorithm still holds a significant advantage over other algorithms. Finally, the results from the DNME3 100 dataset showed that Epi-SSA has a considerable advantage in detecting higher-order epistatic interactions compared to other algorithms. After a multitude of simulation experiments, we are confident that Epi-SSA is an extremely useful algorithm for detecting epistatic interactions in GWAS data, particularly adept at detecting higher-order epistasis.

Furthermore, Epi-SSA was utilized to detect epistasis on a real GWAS dataset of seven complex diseases. It detected a significant number of epistatic interactions related to the seven complex diseases in the dataset and constructed SNP and gene networks for the results. We believe these findings are of significant importance for further exploration of these seven complex diseases.

## Supporting information

S1 Fig Power comparisons between AntEpiSeeker(A), DECMDR(D), HS-MMGKG(G), SEE(S), SHEIB-AGM(B), SNPHarvester(H), SNPRuler(R) and Epi-SSA(P) on the DME 100 dataset. (PDF)

S2 Fig Execution time comparisons between AntEpiSeeker(A), DECMDR(D), HS-MMGKG(G), SEE(S), SHEIB-AGM(B), SNPHarvester(H), SNPRuler(R) and Epi-SSA(P) on the DME 100 dataset. (PDF)

S3 Fig Power comparisons between AntEpiSeeker(A), DECMDR(D), HS-MMGKG(G), SEE(S), SHEIB-AGM(B), SNPHarvester(H), SNPRuler(R) and Epi-SSA(P) on the DNME 100 dataset. (PDF)

S4 Fig Execution time comparisons between AntEpiSeeker(A), DECMDR(D), HS-MMGKG(G), SEE(S), SHEIB-AGM(B), SNPHarvester(H), SNPRuler(R) and Epi-SSA(P) on the DNME 100 dataset. (PDF)

S5 Fig Power comparisons between AntEpiSeeker(A), DECMDR(D), HS-MMGKG(G), SEE(S), SHEIB-AGM(B), SNPHarvester(H), SNPRuler(R) and Epi-SSA(P) on the DME 1000 dataset. (PDF)

S6 Fig Execution time comparisons between AntEpiSeeker(A), DECMDR(D), HS-MMGKG(G), SEE(S), SHEIB-AGM(B), SNPHarvester(H), SNPRuler(R) and Epi-SSA(P) on the DME 1000 dataset. (PDF)

S7 Fig Power comparisons between AntEpiSeeker(A), DECMDR(D), HS-MMGKG(G), SEE(S), SHEIB-AGM(B), SNPHarvester(H), SNPRuler(R) and Epi-SSA(P) on the DNME 1000 dataset. (PDF)

S8 Fig Execution time comparisons between AntEpiSeeker(A), DECMDR(D), HS-MMGKG(G), SEE(S), SHEIB-AGM(B), SNPHarvester(H), SNPRuler(R) and Epi-SSA(P) on the DNME 1000 dataset. (PDF)

S9 Fig Power comparisons between DECMDR(D), HS-MMGKG(G), SEE(S), SHEIB-AGM(B) and Epi-SSA(P) on the DNME3 100 dataset. (PDF)

S10 Fig Execution time comparisons between DECMDR(D), HS-MMGKG(G), SEE(S), SHEIB-AGM(B) and Epi-SSA(P) on the DNME3 100 dataset. (PDF)

S11 Fig The SNP network of the epistatic interactions detected for Coronary Artery Disease (only including SNP pairs with occurrences not less than 60). (PDF)

S12 Fig The SNP network of the epistatic interactions detected for Crohn’s Disease (only including SNP pairs with occurrences not less than 4). (PDF)

S13 Fig The SNP network of the epistatic interactions detected for Hypertension (only including SNP pairs with occurrences not less than 4). (PDF)

S14 Fig The SNP network of the epistatic interactions detected for Rheumatoid Arthritis (only including SNP pairs with occurrences not less than 20). (PDF)

S15 Fig The SNP network of the epistatic interactions detected for Type 1 Diabetes (only including SNP pairs with occurrences not less than 20). (PDF)

S16 Fig The SNP network of the epistatic interactions detected for Type 2 Diabetes (only including SNP pairs with occurrences not less than 4). (PDF)

S17 Fig The gene network of the epistatic interactions detected for Coronary Artery Disease (only including gene pairs with occurrences not less than 60). (PDF)

S18 Fig The gene network of the epistatic interactions detected for Crohn’s Disease (only including gene pairs with occurrences not less than 4). (PDF)

S19 Fig The gene network of the epistatic interactions detected for Hypertension(only including gene pairs with occurrences not less than 4). (PDF)

S20 Fig The gene network of the epistatic interactions detected for Rheumatoid Arthritis(only including gene pairs with occurrences not less than 20). (PDF)

S21 Fig The gene network of the epistatic interactions detected for Type 1 Diabetes(only including gene pairs with occurrences not less than 20). (PDF)

S22 Fig The gene network of the epistatic interactions detected for Type 2 Diabetes (only including gene pairs with occurrences not less than 4). (PDF)

S1 Table The penetrance tables for the 8 DME models. (XLSX)

S2 Table The penetrance tables for the 8 DNME models. (XLSX)

S3 Table The penetrance tables for the 6 DNME3 models. (XLSX)

S4 Table F-measure comparisons between AntEpiSeeker(A), DECMDR(D), HS-MMGKG(G), SEE(S), SHEIB-AGM(B), SNPHarvester(H), SNPRuler(R) and Epi-SSA(P) on the DME 100 dataset. (XLSX)

S5 Table Power comparisons between AntEpiSeeker(A), DECMDR(D), HS-MMGKG(G), SEE(S), SHEIB-AGM(B), SNPHarvester(H), SNPRuler(R) and Epi-SSA(P) on the DME 100 dataset. (XLSX)

S6 Table Execution time comparisons between AntEpiSeeker(A), DECMDR(D), HS-MMGKG(G), SEE(S), SHEIB-AGM(B), SNPHarvester(H), SNPRuler(R) and Epi-SSA(P) on the DME 100 dataset. (XLSX)

S7 Table F-measure comparisons between AntEpiSeeker(A), DECMDR(D), HS-MMGKG(G), SEE(S), SHEIB-AGM(B), SNPHarvester(H), SNPRuler(R) and Epi-SSA(P) on the DNME 100 dataset. (XLSX)

S8 Table Power comparisons between AntEpiSeeker(A), DECMDR(D), HS-MMGKG(G), SEE(S), SHEIB-AGM(B), SNPHarvester(H), SNPRuler(R) and Epi-SSA(P) on the DNME 100 dataset. (XLSX)

S9 Table Execution time comparisons between AntEpiSeeker(A), DECMDR(D), HS-MMGKG(G), SEE(S), SHEIB-AGM(B), SNPHarvester(H), SNPRuler(R) and Epi-SSA(P) on the DNME 100 dataset. (XLSX)

S10 Table F-measure comparisons between AntEpiSeeker(A), DECMDR(D), HS-MMGKG(G), SEE(S), SHEIB-AGM(B), SNPHarvester(H), SNPRuler(R) and Epi-SSA(P) on the DME 1000 dataset. (XLSX)

S11 Table Power comparisons between AntEpiSeeker(A), DECMDR(D), HS-MMGKG(G), SEE(S), SHEIB-AGM(B), SNPHarvester(H), SNPRuler(R) and Epi-SSA(P) on the DME 1000 dataset. (XLSX)

S12 Table Execution time comparisons between AntEpiSeeker(A), DECMDR(D), HS-MMGKG(G), SEE(S), SHEIB-AGM(B), SNPHarvester(H), SNPRuler(R) and Epi-SSA(P) on the DME 1000 dataset. (XLSX)

S13 Table F-measure comparisons between AntEpiSeeker(A), DECMDR(D), HS-MMGKG(G), SEE(S), SHEIB-AGM(B), SNPHarvester(H), SNPRuler(R) and Epi-SSA(P) on the DNME 1000 dataset. (XLSX)

S14 Table Power comparisons between AntEpiSeeker(A), DECMDR(D), HS-MMGKG(G), SEE(S), SHEIB-AGM(B), SNPHarvester(H), SNPRuler(R) and Epi-SSA(P) on the DNME 1000 dataset. (XLSX)

S15 Table Execution time comparisons between AntEpiSeeker(A), DECMDR(D), HS-MMGKG(G), SEE(S), SHEIB-AGM(B), SNPHarvester(H), SNPRuler(R) and Epi-SSA(P) on the DNME 1000 dataset. (XLSX)

S16 Table F-measure comparisons between DECMDR(D), HS-MMGKG(G), SEE(S), SHEIB-AGM(B) and Epi-SSA(P) on the DNME3 100 dataset. (XLSX)

S17 Table Power comparisons between DECMDR(D), HS-MMGKG(G), SEE(S), SHEIB-AGM(B) and Epi-SSA(P) on the DNME3 100 dataset. (XLSX)

S18 Table Execution time comparisons between DECMDR(D), HS-MMGKG(G), SEE(S), SHEIB-AGM(B) and Epi-SSA(P) on the DNME3 100 dataset. (XLSX)

S19 Table The brief description on the WTCCC dataset. (XLSX)

S20 Table Epistatic interactions found by Epi-SSA (n = 600 maxG = 800000). (XLSX)

S21 Table Gene pairs of the epistastic interactions detected on the seven GWAS data using Epi-SSA. (XLSX)

S22 Table Genes of the epistastic interactions detected on the seven GWAS data using Epi-SSA. (XLSX)
