# Supplementary material for: Epi-SSA: A novel epistasis detection method based on a multi-objective sparrow search algorithm
Source: PLoS One. 2024 Oct 24;19(10):e0311223. doi: 10.1371/journal.pone.0311223 (PMC11500897; doi:10.1371/journal.pone.0311223)
Supplement: S5 Fig — (PDF) [file pone.0311223.s005.pdf]

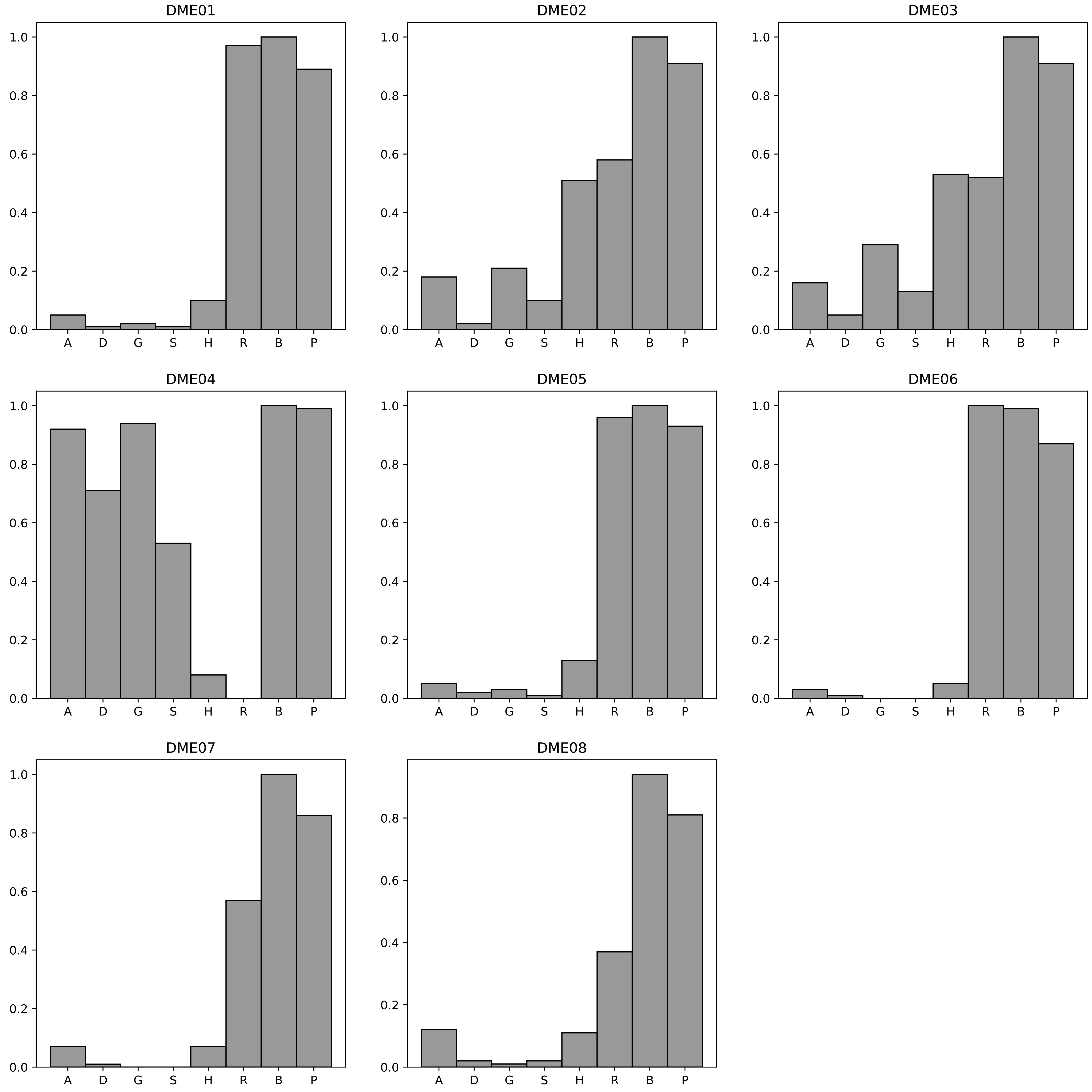

Fig S5. Power comparisons between AntEpiSeeker(A), DECMDR(D), HS-MMGKG(G), SEE(S), SHEIB-AGM(B), SNPHarvester(H), SNPRuler(R) and Epi-SSA(P) on the DME 1000 dataset.
