# Supplementary figures and images for: Epi-SSA: A novel epistasis detection method based on a multi-objective sparrow search algorithm
Source: PLoS One. 2024 Oct 24;19(10):e0311223. doi: 10.1371/journal.pone.0311223 (PMC11500897; doi:10.1371/journal.pone.0311223)

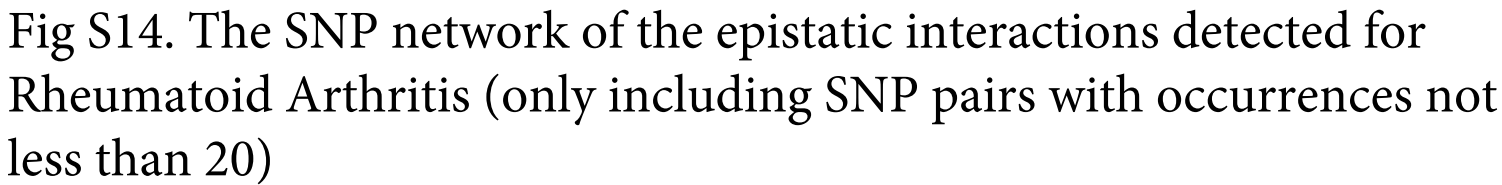

Supplement: S14 Fig — (PDF) [file pone.0311223.s014.pdf]

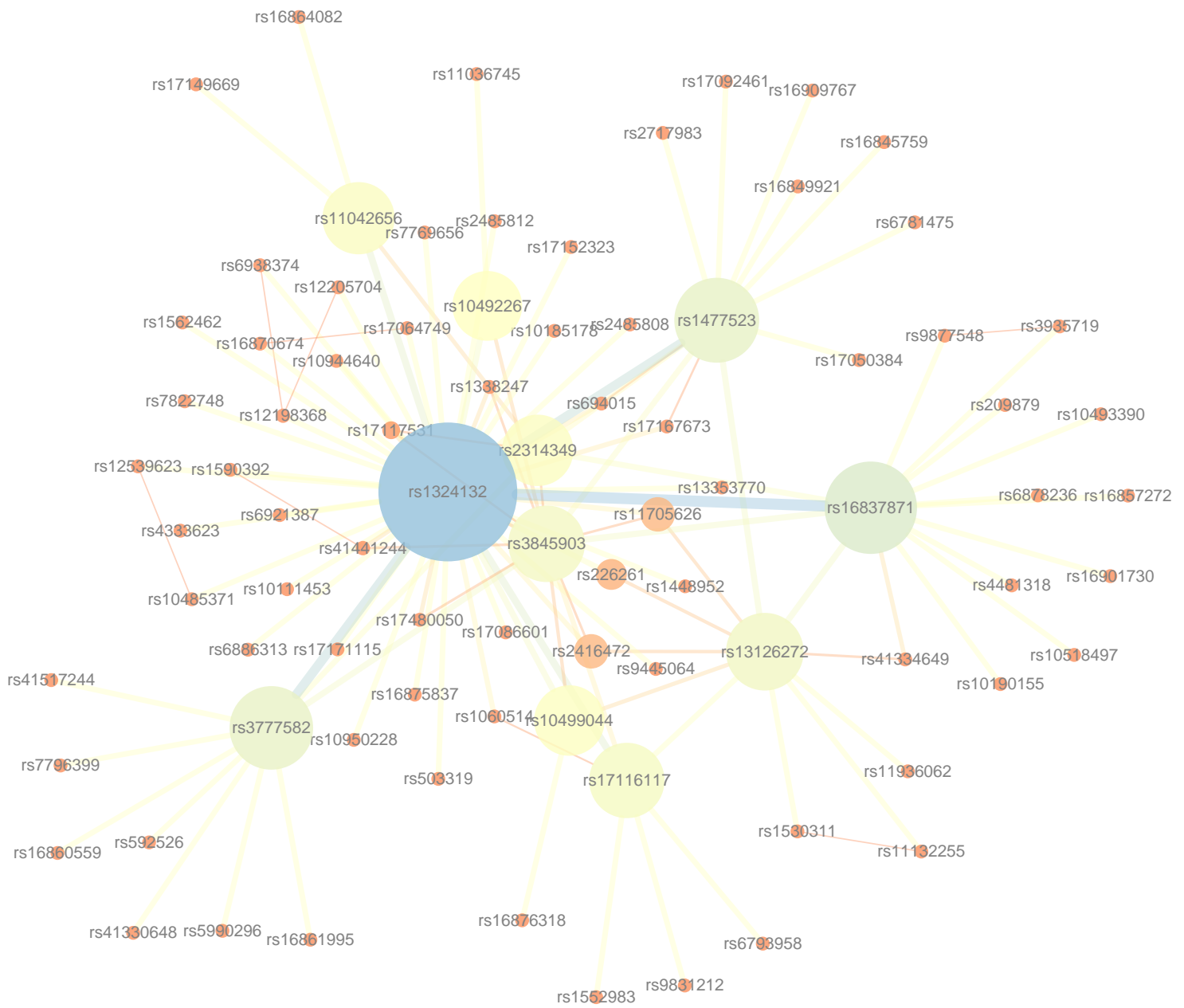

Supplement: S16 Fig — (PDF) [file pone.0311223.s016.pdf]

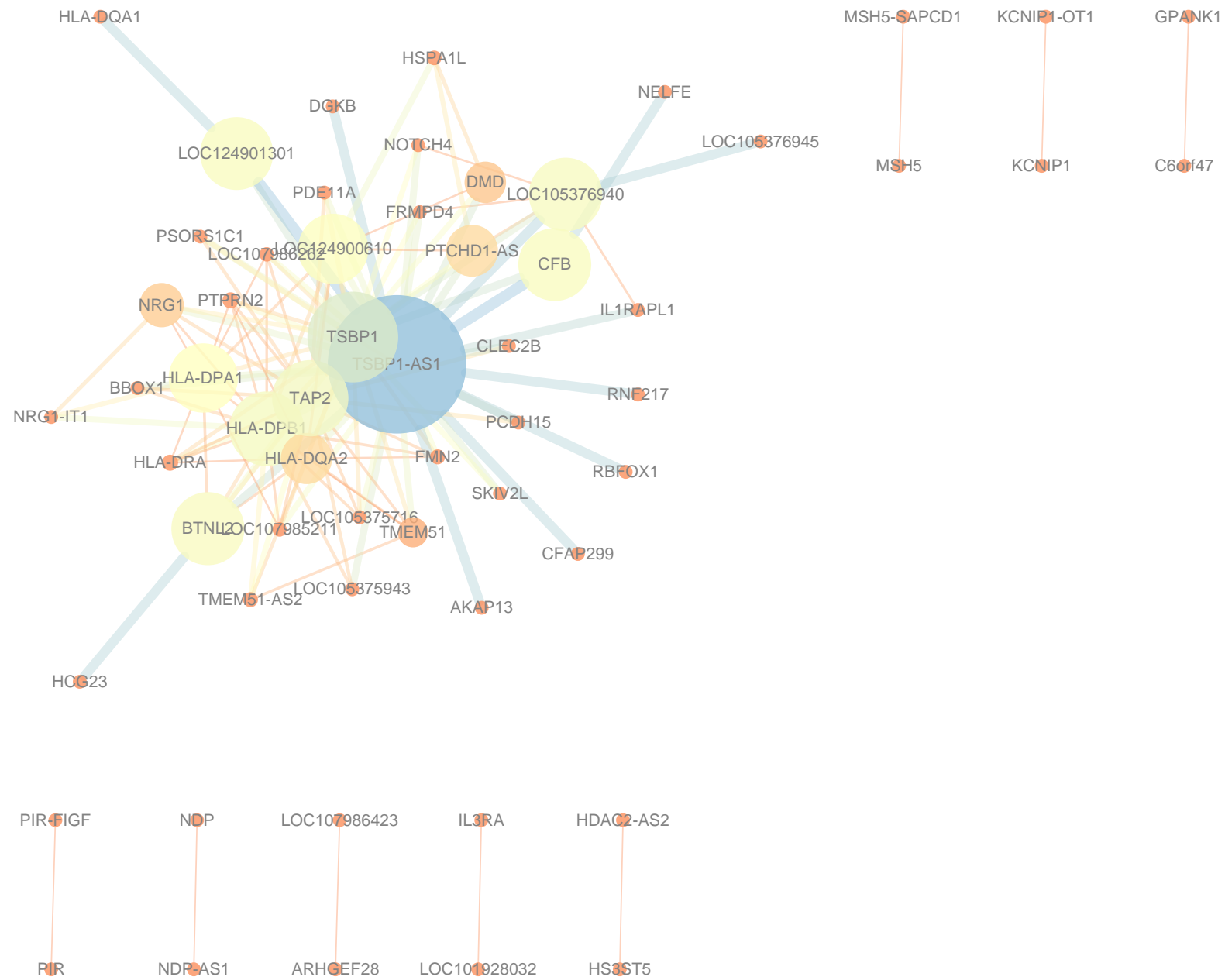

Supplement: S20 Fig — (PDF) [file pone.0311223.s020.pdf]
