# Supplementary material for: Epi-SSA: A novel epistasis detection method based on a multi-objective sparrow search algorithm
Source: PLoS One. 2024 Oct 24;19(10):e0311223. doi: 10.1371/journal.pone.0311223 (PMC11500897; doi:10.1371/journal.pone.0311223)
Supplement: S22 Fig — (PDF) [file pone.0311223.s022.pdf]

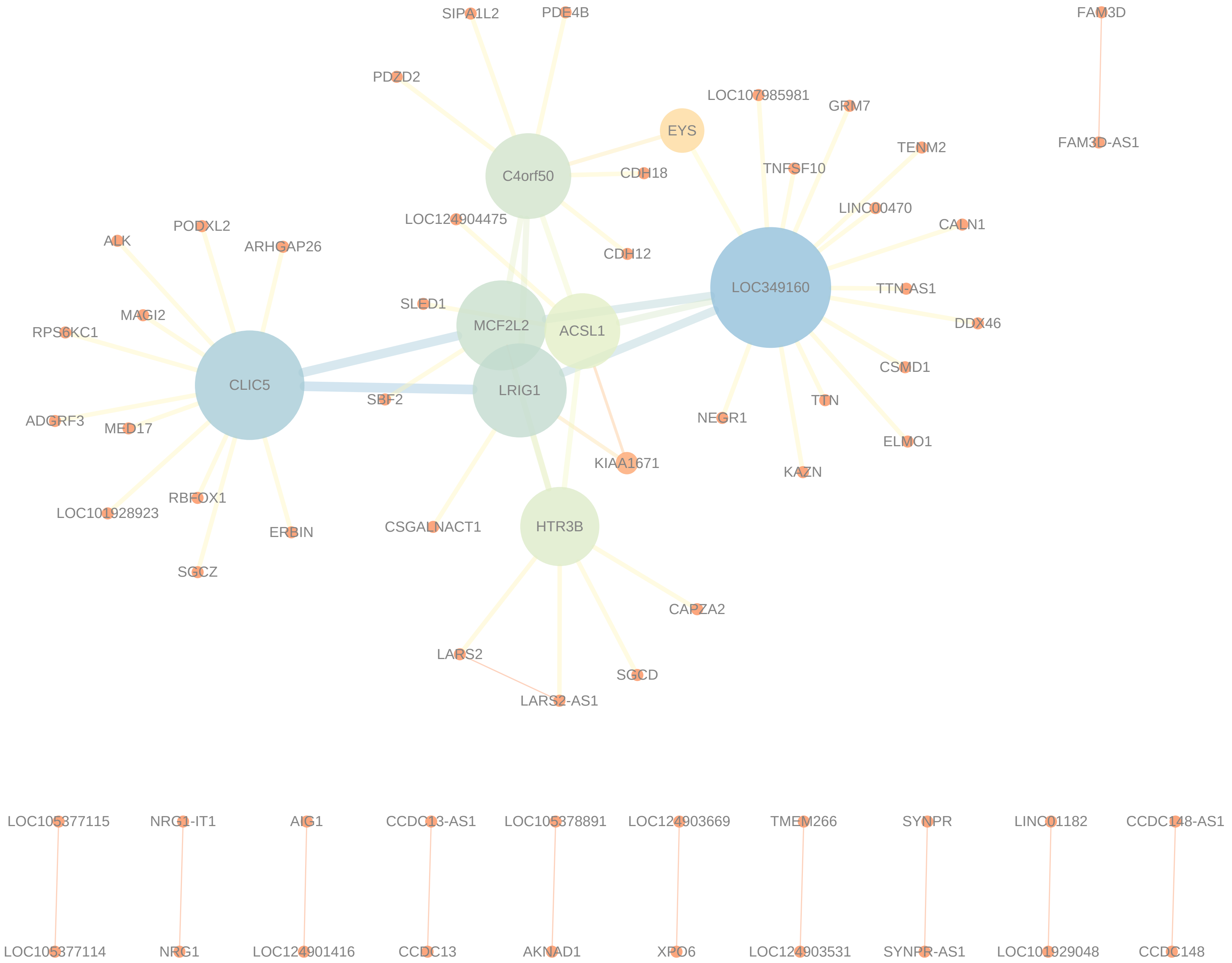

Fig S22. The gene network of the epistatic interactions detected for Type 2 Diabetes (only including gene pairs with occurrences not less than 4)
